# The protein kinases KIPK and KIPK-LIKE1 suppress overbending during negative hypocotyl gravitropic growth in Arabidopsis

**DOI:** 10.1093/plcell/koaf056

**Published:** 2025-04-22

**Authors:** Yao Xiao, Melina Zourelidou, Alkistis E Lanassa Bassukas, Benjamin Weller, Dorina P Janacek, Jan Šimura, Karin Ljung, Ulrich Z Hammes, Jia Li, Claus Schwechheimer

**Affiliations:** Plant Systems Biology, School of Life Sciences, Technical University of Munich, Emil-Ramann-Strasse 8, Freising 85354, Germany; Guangdong Provincial Key Laboratory of Plant Adaptation and Molecular Design, School of Life Sciences, Guangzhou University, Guangzhou 510006, China; Plant Systems Biology, School of Life Sciences, Technical University of Munich, Emil-Ramann-Strasse 8, Freising 85354, Germany; Plant Systems Biology, School of Life Sciences, Technical University of Munich, Emil-Ramann-Strasse 8, Freising 85354, Germany; Plant Systems Biology, School of Life Sciences, Technical University of Munich, Emil-Ramann-Strasse 8, Freising 85354, Germany; Plant Systems Biology, School of Life Sciences, Technical University of Munich, Emil-Ramann-Strasse 8, Freising 85354, Germany; Department of Forest Genetics and Plant Physiology, Umeå Plant Science Centre, Swedish University of Agricultural Sciences, 90736 Umeå, Sweden; Department of Forest Genetics and Plant Physiology, Umeå Plant Science Centre, Swedish University of Agricultural Sciences, 90736 Umeå, Sweden; Plant Systems Biology, School of Life Sciences, Technical University of Munich, Emil-Ramann-Strasse 8, Freising 85354, Germany; Guangdong Provincial Key Laboratory of Plant Adaptation and Molecular Design, School of Life Sciences, Guangzhou University, Guangzhou 510006, China; Plant Systems Biology, School of Life Sciences, Technical University of Munich, Emil-Ramann-Strasse 8, Freising 85354, Germany

## Abstract

Plants use environmental cues to orient organ and plant growth, such as the direction of gravity or the direction, quantity, and quality of light. During the germination of *Arabidopsis thaliana* seeds in soil, negative gravitropism responses direct hypocotyl elongation such that the seedling can reach the light for photosynthesis and autotrophic growth. Similarly, hypocotyl elongation in the soil also requires mechanisms to efficiently grow around obstacles such as soil particles. Here, we identify KIPK (KINESIN-LIKE CALMODULIN-BINDING PROTEIN-INTERACTING PROTEIN KINASE) and the paralogous KIPKL1 (KIPK-LIKE1) as genetically redundant regulators of gravitropic hypocotyl bending. Moreover, we demonstrate that the homologous KIPKL2 (KIPK-LIKE2), which shows strong sequence similarity, must be functionally distinct. KIPK and KIPKL1 are polarly localized plasma membrane-associated proteins that can activate PIN-FORMED auxin transporters. KIPK and KIPKL1 are required to efficiently align hypocotyl growth with the gravity vector when seedling hypocotyls are grown on media plates or in soil, where contact with soil particles and obstacle avoidance impede direct negative gravitropic growth. Therefore, the polar KIPK and KIPKL1 kinases have different biological functions from the related AGC1 family kinases D6PK (D6 PROTEIN KINASE) or PAX (PROTEIN KINASE ASSOCIATED WITH BRX).

## Teaser

Protein kinases are essential for enabling seedlings to grow against gravity, potentially by facilitating the appropriate distribution of auxin throughout the growing seedling hypocotyl.

## Introduction

Plants use environmental cues, such as the direction of gravity or the direction, quantity, and quality of light, to orient organ and plant growth. During germination of *Arabidopsis thaliana* seeds in the soil, hypocotyl elongation is directed by negative gravitropism. Hypocotyl elongation in the soil, however, also requires mechanisms to efficiently grow around obstacles that impede elongation through the soil (Britz and Galston 1982; Takahashi and Jaffe 1990; Takahashi et al. 2000; Gupta et al. 2012). Using the limited resources of the seed, seedling hypocotyls must efficiently combine gravitropic growth and bending responses to reach sunlight for photomorphogenic growth and photosynthesis (Jonsson et al. 2023). Only little is known about how germinating seedlings integrate these responses to direct the hypocotyl through the soil.

The phytohormone auxin regulates many aspects of plant growth and development (Teale et al. 2006; Weijers et al. 2022). During the directed tropic growth of roots and shoots, differential auxin distribution is commonly observed in the bending tissue, e.g. during negative gravitropic growth, during phototropic bending, as well as during thigmotropic responses, possibly to mediate cell wall loosening and turgor-driven cell elongation (Rakusova et al. 2011, 2016; Grones et al. 2018; Lee et al. 2020; Han et al. 2021; Hajny et al. 2022). Indole-3-acetic acid (IAA), the most abundant auxin in land plants, is transported from cell to cell by auxin efflux and influx carriers (Hammes et al. 2022). “Canonical” PIN-FORMED (PIN) auxin transporters are polarly distributed in the plasma membranes of many cells, and their polar distribution allows predicting auxin transport through tissues, which may serve to explain auxin-dependent growth, differentiation, and tropisms (Teale et al. 2006; Rakusova et al. 2011, 2016; Grones et al. 2018; Sauer and Kleine-Vehn 2019). Tropic root and shoot bending are associated with the differential accumulation of auxin, the presumed result of differential PIN auxin transporter distribution (Friml et al. 2002; Abas et al. 2006). Specifically, *pin2 A. thaliana* mutants have agravitropically growing roots (Luschnig et al. 1998), while triple mutants of the paralogous *PIN3*, *PIN4*, and *PIN7* (*pin347*) are severely impaired in hypocotyl phototropism and negative gravitropism (Willige et al. 2013). In the hypocotyl, the bending response during negative gravitropism has been explained by the redistribution of PIN3 towards the lower side of the hypocotyl endodermis to allow for auxin accumulation and enhanced cell elongation in the lower side of the hypocotyl (Rakusova et al. 2011). This redistribution is reversed, and the bending response is terminated when the optimal angle is reached, presumably as a consequence of the resulting increased cellular auxin accumulation (Rakusova et al. 2016; Grones et al. 2018).

Some members of the ABCB family act as plasma membrane-resident auxin transporters (Noh et al. 2003; Geisler et al. 2005, 2017; Hammes et al. 2022; Ying et al. 2024). One member of this family, ABCB19, has recently been reported to transport brassinosteroid hormones (Ying et al. 2024). Importantly, single and double mutants of the ABCB transporters *ABCB1/PGP1* and *ABCB19/MDR1/PGP19* show exaggerated hypocotyl bending responses, suggesting that, in the absence of these transporters, the resulting differential auxin or, as more recent findings suggest, brassinosteroid distribution impairs normal tropic growth (Noh et al. 2003).

PIN transporters are activated by AGCVIII family kinases, plant-specific serine/threonine kinases that can be identified by an insertion between protein kinase subdomains VII and VIII (Galvan-Ampudia and Offringa 2007; Bassukas et al. 2022). In *A. thaliana*, the AGCVIII kinases phototropin1 (phot1) and phot2, which form the AGC4 clade of the AGCVIII kinases, are blue light receptor kinases that promote phototropic hypocotyl bending towards the light (Briggs and Christie 2002). Furthermore, several AGC1 and AGC3 kinases, namely the AGC1 kinases D6 PROTEIN KINASE (D6PK) together with the related D6PK-LIKE1 (D6PKL1)—D6PKL3, as well as the AGC3 kinases PINOID (PID), WAG1, and WAG2, have been implicated in the regulation of photo- and/or gravitropism responses (Sukumar et al. 2009; Rahman et al. 2010; Ding et al. 2011; Willige et al. 2013; Haga et al. 2014). All of the mentioned AGC1 and AGC3 kinases are localized at the plasma membrane, where they activate PINs and auxin transport by phosphorylation (Barbosa et al. 2014; Zourelidou et al. 2014). AGC1, but not AGC3 kinases, are polarly localized, like PINs, but their transport to and from the plasma membrane as well as the mechanisms controlling their plasma membrane polarity are distinct from those of PINs (Barbosa et al. 2014, 2016; Graf et al. 2024). D6PK and PID plasma membrane interactions are mediated by interactions between polybasic stretches in the kinase insertion domain and anionic phospholipids in the plasma membrane, as well as, in the case of D6PK, S-acylation at conserved repeated CXX(X)P motifs and a phosphoregulation in the insertion domain (Barbosa et al. 2016; Simon et al. 2016; Graf et al. 2024). The biological functions of several AGC1 kinases remain to be established.

Here, we characterize the *A. thaliana* AGC1 kinases KIPK (KINESIN-LIKE CALMODULIN-BINDING PROTEIN INTERACTING PROTEIN KINASE, AT3G52890) and its 2 paralogues KIPKL1/AGC1.9 (KIPK-LIKE1; AT2G36350) and KIPKL2/AGC1.8 (KIPK-LIKE1; AT5G03640; Galvan-Ampudia and Offringa 2007). KIPK was originally identified as a yeast 2-hybrid interactor of ZWI/KINESIN-LIKE CALMODULIN-BINDING PROTEIN (AT5G65930), but no relevant phenotype has been described with regard to ZWI function in trichome differentiation (Day et al. 2000). An independent study identified KIPK and KIPKL1 as yeast 2-hybrid interactors of the putative cell wall-sensing PROLINE-RICH, EXTENSION-LIKE RECEPTOR-LIKE KINASE (PERK10, AT1G26150) and its close homologs, and mild root growth defects were reported when seedlings were grown on media with high sucrose content (Humphrey et al. 2015). While it is known that the *KIPK* ortholog *DWARF2* (*DW2*) from *Sorghum bicolor* regulates stem internode length (Hilley et al. 2017), as yet, no major biological function has been associated with KIPK/KIPKL proteins in *A. thaliana*.

Our study shows that *kipk kipkl1* mutants exhibit exaggerated negative hypocotyl gravitropism when mutants are grown vertically along media and overbending when seedling hypocotyls grow through soil. We further show that *KIPKL2* does not contribute to this phenotype and cannot replace *KIPK* and *KIPKL1*. Mutants of the reported KIPK interactors *KINESIN-LIKE CALMODULIN-BINDING PROTEIN/ZWICHEL* (*ZWI*) and *PERK* (*PROLINE-RICH EXTENSION-LIKE RECEPTOR-LIKE KINASE*) did not show tropism defects and are unlikely to be involved in negative hypocotyl gravitropism and obstacle avoidance growth examined here. We thereby uncover a biological function for KIPK family AGC1 kinases that is functionally distinct from other AGC1 kinase family members such as D6PK and PAX.

## Results

### KIPK, KIPKL1, and KIPKL2 are polarly localized plasma membrane-associated protein kinases

*Arabidopsis thaliana KIPK* and the 2 closely related *KIPKL1* and *KIPKL2* have unknown biological functions. When compared with other AGC1 kinases, including the well-studied D6PK (108 amino acids), these 3 kinases have extended (537 to 558 amino acids) homologous (27% to 63%) N-termini that are reminiscent of the long N-termini of the AGC4 kinases phot1 and phot2 (576 to 662 amino acids; Supplementary Fig. S1). While these extended N-termini do not contain motifs or domains of known or predictable function, the presence of CXX(X)P repeats in the middle domain and polybasic regions, which, in D6PK, have been shown to function for S-acylation and interactions with plasma membrane phospholipids, respectively, suggests that they may be plasma membrane-associated protein kinases (Barbosa et al. 2016; Bassukas et al. 2022; Graf et al. 2024).

Promoter expression analysis of 2 kb *KIPK*, *KIPKL1*, and *KIPKL2* promoter fragments with the GUS (ß-glucuronidase) reporter indicated that all 3 genes are broadly expressed in dark- and light-grown seedlings and leaves (Supplementary Fig. S2). Noteworthy is the speckled staining in root tips, obtained with pKIPKL2::GUS, which is indicative of cell cycle-dependent expression (Supplementary Fig. S2C).

**Figure 1.**
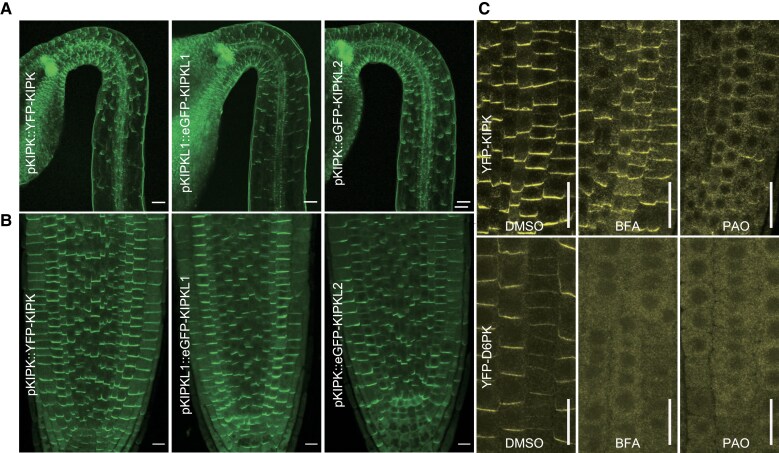
KIPK, KIPKL1, and KIPKL2 are polarly localized plasma membrane-associated protein kinases. **A)** and **B)** Representative confocal images of the epidermal cells of hypocotyls with their apical hooks **A)** and the root tips **B)** from 3-d-old dark-grown seedlings expressing the fluorescent protein-tagged KIPK/KIPKLs as specified in the figure panels. Scale bars = 50 *µ*m **A)** and 10 *µ*m **B)**. **C)** Representative confocal images of root epidermal cells expressing YFP-KIPK and YFP-D6PK following a 30 min mock (DMSO), 25 *µ*M BFA (Brefeldin A) or 30 *µ*M PAO (phenylarsine oxide) treatment. Note that while YFP-D6PK dissociates fully from the plasma membrane after BFA treatment, YFP-KIPK dissociates only partially under these conditions. Scale bar = 20 *µ*m.

To examine the subcellular localization of the KIPK and KIPKL proteins, we expressed YFP- or eGFP-tagged variants from the 2 kb *pKIPK* or *pKIPKL1* promoter fragment from pKIPK::YFP-KIPK, pKIPKL1::eGFP-KIPKL1 or pKIPK::eGFP-KIPKL2. KIPK and the KIPKLs localized polarly at the basal (rootward) plasma membrane in all cells examined, e.g. in all cells of the seeding hypocotyl and root epidermis (Fig. 1, A and B). The related D6PK is rapidly cycling to and from the plasma membrane, which can be visualized through treatments with Brefeldin A (BFA), an inhibitor of GNOM family guanine exchange factors (GEFs; Fig. 1C; Barbosa et al. 2014). In the case of KIPK, BFA treatment (30 min) had a comparatively small effect on YFP-KIPK cellular distribution, indicating that KIPK trafficking, unlike D6PK trafficking, may be largely independent from BFA-sensitive GNOM family members or may be slower than D6PK trafficking (Fig. 1C).

### KIPK, KIPKL1, and KIPKL2 bind anionic phospholipids

Like D6PK and all other AGC1 kinases, KIPK and KIPKLs contain an insertion domain between protein kinase subdomains VII and VIII, which, in the case of D6PK, contains sequence motifs for D6PK plasma membrane association, D6PK recycling, and polarity maintenance (Supplementary Fig. S1; Bassukas et al. 2022; Graf et al. 2024). E.g., the D6PK insertion domain contains a polybasic motif required for interactions with negatively charged phospholipids at the plasma membrane and during vesicular trafficking (Barbosa et al. 2016). In experiments with phospholipid-containing PIP strips, recombinant KIPK and KIPKLs bound to mono-, di-, and tri-phosphorylated phosphoinositides, as well as to phosphatidic acid, and thereby displayed a similar phosphoinositide preference as D6PK (Supplementary Fig. S3A; Barbosa et al. 2016). Following treatments with a panel of phosphoinositide biosynthesis inhibitors, we found that PAO (phenylarsine oxide), an inhibitor of PtdIns4P (phosphoinositol-4-phosphate) synthesis, causes dissociation of YFP-KIPK from the plasma membrane (Fig. 1C, Supplementary Fig. S3B and C; Simon et al. 2016). Thereby, YFP-KIPK displayed a similar inhibitor sensitivity and behavior as YFP-D6PK with regard to PAO but responded differently to U73122, 1-butanol, and FIPI, whose effects on YFP-D6PK are, however, also less pronounced (Fig. 1C; Supplementary Fig. S3, B and C). We concluded that KIPK required PtdIns4P or its phosphorylated derivatives for interactions with the plasma membrane.

Regions enriched in the basic amino acids arginine (R) and lysine (K), e.g. regions with a basic hydrophobicity (BH) score greater than 0.6, are proposed phospholipid interaction domains of membrane-associated proteins (Bailey and Prehoda 2015). We identified a highly basic and conserved region in the N-termini of KIPK and KIPKL1 (Supplementary Fig. S4, A and B; Bassukas et al. 2022). In this regard, KIPK and KIPKL1 differ from D6PK, which contains a phospholipid-binding polybasic region within the insertion domain (Supplementary Fig. S4, A and C; Barbosa et al. 2016; Bassukas et al. 2022). Intriguingly, KIPKL2 contained a basic region in the insertion domain, which in turn was absent from KIPK or KIPKL1 (Supplementary Fig. S4, A and C).

We examined the relevance of the basic region from KIPK for plasma membrane interaction using a KIPK mutant variant where 12 K and R residues were replaced by the uncharged alanine (KR12A; Supplementary Fig. S4B). When expressed as a YFP-fusion, KIPK-YFP (KR12A) localized polarly at the basal plasma membrane and was, in this regard, indistinguishable from wild-type YFP-KIPK (Supplementary Fig. S4D). We concluded that the highly basic region at the KIPK N-terminus is not required for plasma membrane interactions, at least not when tested in isolation.

### KIPK and KIPKL1 activate PIN-mediated auxin export in *Xenopus laevis* oocytes

**Figure 2.**
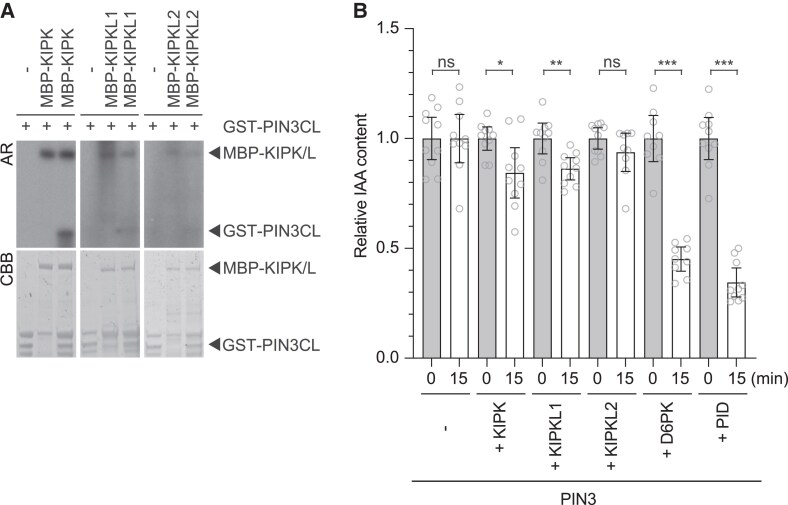
KIPK and KIPKL1 phosphorylate PIN3 and activate PIN3-mediated auxin transport. **A)** Autoradiographs (AR) and Coomassie Brilliant Blue- (CBB-)stained gels (loading control) from in vitro phosphorylation experiments with recombinant purified MBP (maltose binding protein)-KIPK and MBP-KIPKLs (MBP-KIPK/L) and the GST (glutathione-S-transferase)-tagged cytoplasmic loop of PIN3 (GST-PIN3CL). **B)** Results from auxin transport experiments with PIN3 and protein kinases as specified. Shown is the IAA (indole-3-acetic acid) content after injection (grey bars) set to 1 in comparison to the IAA content after 15 min of efflux (white bars) expressed relative to the starting content. Shown are the individual data points from at least 9 individual oocytes, means and standard errors. Groups were compared by a Student's *t*-test: *P < 0.05; **P < 0.01; ***P < 0.001; ns, not significant.

AGC1 (D6PK, PAX) and AGC3 (PID, WAG2) kinases phosphorylate the cytoplasmic loops of “long” PINs and activate PIN-mediated auxin transport from *Xenopus laevis* oocytes (Zourelidou et al. 2014; Marhava et al. 2018). Similarly, recombinant purified MBP- (maltose binding protein-) tagged MBP-KIPK and MBP-KIPKL1 phosphorylated the GST- (glutathione-S-transferase-) tagged PIN3 cytoplasmic loop (GST-PIN3CL) in vitro, while GST-PIN3CL phosphorylation by MBP-KIPKL2 was reproducibly comparatively less efficient than phosphorylation by the other 2 kinases (Fig. 2A).

In *Xenopus laevis* oocytes, PIN3-mediated auxin efflux was activated when *PIN3* was co-expressed with *KIPK* or *KIPKL1* but not significantly when co-expressed with *KIPKL2*, suggesting that KIPK and KIPKL1, like D6PK, PAX, or PID, are activators of PIN-mediated auxin efflux (Fig. 2B; Zourelidou et al. 2014; Marhava et al. 2018). In comparison with D6PK and PID, which were positive controls in our experiment, PIN3 activation by KIPK and KIPKL1 was less efficient, which may be due to differences in kinase activity and activation, protein expression in the oocyte system, or, alternatively, a reflection of true biologically relevant differences.

### *Kipk01* and *kipk012* mutants display an exaggerated negative gravitropism response

To characterize the biological function of the KIPK and KIPKLs, we isolated homozygous single mutants, *kipk0*, *kipkl1-1*, *kipk1-2*, and *kipkl2*, and generated *kipk01* (*kipk0 kipkl1-1*), *kipk01-2* (*kipk0 kipkl1-2*), *kipk02* (*kipk0 kipkl2*), and *kipk12* (*kipk1-1 kipkl2*) double mutants, as well as a *kipk012* (*kipk0 kipk1-1 kipkl2*) triple mutant by genetic crosses (Supplementary Fig. S5A). When we examined *kipk012* for the presence of transcript fragments spanning the T-DNA insertion, we did not detect *KIPK* or *KIPKL* transcripts, and we concluded that *kipk012* is a loss-of-function mutant of the 3 kinases (Supplementary Fig. S5B).

**Figure 3.**
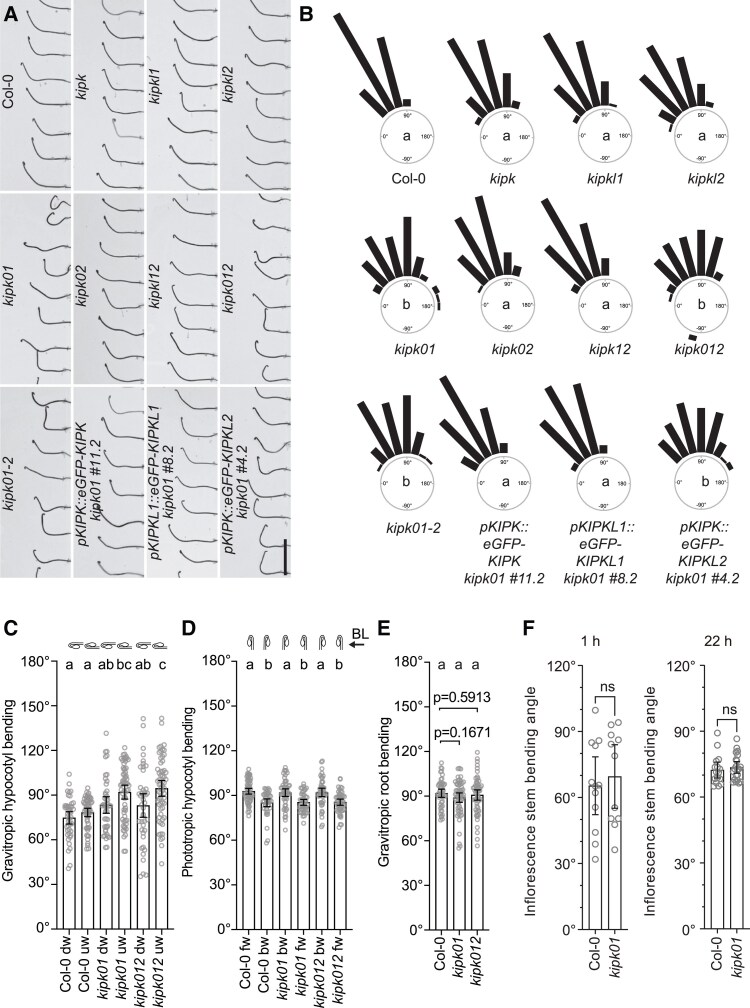
*KIPK* and *KIPKL1* function redundantly in the regulation of negative hypocotyl gravitropism. **A)** Representative photographs of 3-d-old dark-grown seedlings of the specified genotypes 24 h after reorientation by 90°. Scale bar for all panels = 1 cm. **B)** Rose diagrams displaying gravitropic hypocotyl bending angles of seedlings from the experiment shown in **A)**. Seedlings were grouped in 15° angle windows. *n* > 94 seedlings. Statistical significance was assessed using 1-way ANOVA, followed by Dunnett's T3 post hoc test for multiple comparisons. Different letters in the center of each diagram indicate significant differences between groups at *P* < 0.05. *kipk01-2* is a second *kipk01* double mutant combination with the *kipkl1-2* allele whose phenotype is indistinguishable from *kipk01-1* carrying the *kipkl1-1* allele used throughout this study. **C)** to **F)** Graphs displaying the average and 95% confidence interval, as well as the individual data points from a negative hypocotyl gravitropism experiment **C)** as shown in **A)** and **B)**, a hypocotyl phototropism experiment **D)**, a root gravitropism experiment **E)** and a shoot negative gravitropism experiment using main inflorescence stems **F)**. Since it is known that cotyledon positioning influences the degree of hypocotyl bending (Khurana et al. 1989), seedling responses were evaluated independently for seedlings with downward- (dw-) or upward- (uw-) positioned cotyledons **C)** or with forward- (fw-) or backward- (bw-) oriented cotyledons with regard to the gravity vector **C)** or the orientation of the blue light (BL) used for seedling illumination **D)**. *n* > 41 seedlings or *n* ≥ 11 inflorescences. Results from a 1-way ANOVA analysis followed by Dunnett's T3 post hoc test for multiple comparisons or a Welch's *t*-test (*P* > 0.05, ns, not significant) are displayed on the top of each bar. Different letters indicate significant differences between groups at *P* < 0.05.

The *kipk012* triple mutant did not display apparent defects when grown in the light (Supplementary Fig. S5C). Since mutants of other AGCVIII kinases had been implicated in tropic growth responses, we examined gravitropic and phototropic growth in *kipk and kipkl* single, double, and triple mutants. In these experiments, we identified *kipk01* and *kipk012* as mutants with an exaggerated negative gravitropism response in the hypocotyls of dark-grown seedlings when compared to the wild type, the respective single mutants, or the *kipk02* and *kipk12* double mutants (Fig. 3, A and B). Since previous reports had indicated that cotyledon positioning affected the degree of gravitropic response in the hypocotyl, we confirmed and refined mutant phenotyping by resolving gravitropic responses taking into account cotyledon position (Fig. 3C; Khurana et al. 1989). *kipk01* or *kipk012* did not display obvious defects in root gravitropism, phototropic hypocotyl bending, or negative shoot gravitropism (inflorescence stem bending), indicating that the bending defect was, at least among the responses tested, specific for negative hypocotyl bending (Fig. 3, D to F; Supplementary Fig. S5C).

A time-resolved analysis of hypocotyl bending of wild type and *kipk01* mutants showed that the bending response was indistinguishable between the 2 genotypes during the early stages of bending until 8 hrs (Supplementary Fig. S6). After 8 hrs, *kipk01* bent more strongly than the wild type, and bending angles in the *kipk01* mutants were distributed over a broader range of angles than in the wild type (Supplementary Fig. S6). We concluded that the hypocotyl bending defect observed in the *kipk01* mutant was a consequence of increased bending at the later stages of the bending response.

The hypocotyl gravitropism defect of the *kipk01* double mutant was suppressed after expression of *KIPK* or *KIPKL1* from a 2 kb *pKIPK* promoter fragment but not after expression of *KIPKL2* from *pKIPK* (Fig. 3, A and B). This suggested that KIPKL2 was biochemically different from the other 2 family members, a finding also in line with the observation that the loss of *KIPKL2* in the *kipk012* triple mutant did not enhance the hypocotyl gravitropism defect of the *kipk01* double mutant (Fig. 3A). Further, expression of the KIPK(KR12A) variant could still suppress the *kipk012* phenotype, suggesting that the mutated motif is not important for KIPK function (Supplementary Fig. S4E). In summary, we concluded that *KIPK* and *KIPKL1* are required for the regulation of proper negative gravitropism in *A. thaliana* and that *KIPKL2* does not contribute to this process. The observation that *KIPKL2* is unable to complement the *kipk01* phenotype aligned with the differential activity of KIPKL2 in the in vitro phosphorylation and auxin transport experiments (Fig. 2).

### *Kipk01* overbending responses require PIN-mediated auxin transport

**Figure 4.**
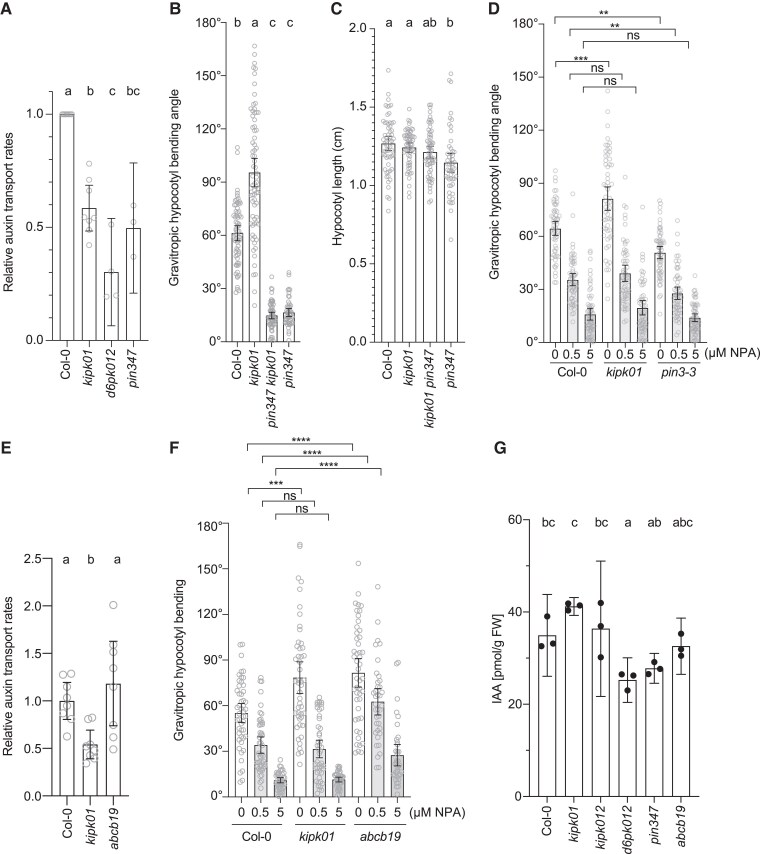
PIN-mediated auxin transport is a prerequisite for *kipk01* hypergravitropic responses. **A)** and **E)** Graphs displaying the average and 95% confidence interval, as well as the individual results, of relative auxin transport rates in wild type (Col-0), *kipk01*, *d6pk012*, and *pin347* mutants; *n* ≥ 3 independent experiments **A)** or *kipk01* and *abcb19* mutants *n* = 8 × 5 seedlings **E)**. The individual results from the auxin transport measurements are shown in Supplementary Table S2. **B)**, **D)**, and **F)** Graphs displaying the average and 95% confidence interval, as well as the individual results, of gravitropic hypocotyl bending experiments with 3-d-old dark-grown wild type (Col-0), *kipk01*, *pin347*, *pin347 kipk01* seedlings **B)**, 3-d-old dark-grown wild type (Col-0), *kipk01* and *pin3-3* seedlings after treatments with NPA **D)**, or with 3-d-old dark-grown wild type (Col-0), *kipk01*, *abcb19* seedlings after treatments with NPA **F)**. *n* > 37 seedlings. Results from a 1-way ANOVA analysis or Welch's *t*-tests are displayed. Groups were compared by a Welch's *t*-test **D, F)**: *P < 0.05; **P < 0.01; ***P < 0.001; ****P < 0.0001; ns, not significant. One-way ANOVA, followed by Tukey's **A)** or Dunnett's T3 **B, E)** post hoc test for multiple comparisons. Different letters indicate significant differences between groups at *P* < 0.05. **C)** Graph displaying the average and 95% confidence interval, as well as the individual results, of hypocotyl lengths measured in 3-d-old dark-grown seedlings of the specified genotypes. Results from a 1-way ANOVA analysis followed by Dunnett's T3 post hoc test for multiple comparisons are displayed. Different letters indicate significant differences between groups at *P* < 0.05. *n* > 49 seedlings. **G)** Graph displaying the average and standard deviation of IAA quantifications in hypocotyls and cotyledons of pools of 4-d-old dark-grown seedlings of the specified genotypes (*n* = 3 pools). Statistical significance was assessed using 1-way ANOVA, followed by Dunnett's T3 post hoc test for multiple comparisons. Different letters indicate significant differences between groups at *P* < 0.05.

The phototropic and gravitropic bending of seedling hypocotyls requires the functionally redundant auxin efflux carriers PIN3, PIN4, and PIN7, as well as their phospho-regulation by D6PK, D6PKL1, and D6PKL2 (Willige et al. 2013). We found that, similar to *d6pk d6pkl1 d6pkl2* (*d6pk012*) or *pin3 pin4 pin7* (*pin347*) triple mutants, *kipk01* mutants displayed a strong reduction in basipetal auxin transport in hypocotyls of dark-grown seedlings, suggesting that KIPK and KIPKL1 participate in basipetal auxin transport in Arabidopsis hypocotyls, possibly as a result of the role of KIPK and KIPKL1 in PIN activation (Fig. 3, Fig. 4A). Since PIN-mediated auxin transport is required for the initial bending response, introducing the *pin347* triple mutant into *kipk01* suppressed the gravitropic bending in kipk01 (Fig. 4B). Importantly, the hypocotyls of dark-grown seedlings of all genotypes had comparable if not identical lengths, indicating that differences in hypocotyl elongation did not interfere with the tropism analyses (Fig. 4C). Following treatments with NPA, an inhibitor of PIN-mediated auxin transport, we found that auxin transport inhibition suppressed the gravitropic responses of the *kipk01* mutants with a similar sensitivity as that observed in the wild type or in *pin3* single mutants, which are only partially impaired in negative gravitropism (Fig. 4D; Abas et al. 2021). These findings are consistent with previously made observations that efficient auxin transport is required for tropic responses but not for hypocotyl elongation in dark-grown seedlings (Jensen et al. 1998). We concluded that PIN-mediated auxin transport is compromised in *kipk01* mutant hypocotyls and that *kipk01* hypocotyl overbending does not supersede the requirement of PIN-mediated auxin transport for bending.

Hypocotyl overbending was previously reported for the ABCB transporter mutant abcb19, whose phenotype is reminiscent of the phenotype of *kipk01* mutants (Noh et al. 2003). In our experimental settings, the *abcb19* mutant had a similar auxin transport comparable to the wild type and had a reduced NPA sensitivity when compared with the wild-type or the *kipk01* mutant (Fig. 4, E and F). The *abcb19* mutant has thus defects in auxin transport that are distinct from those of the *kipk01* or *pin347* mutants. While the auxin (IAA) concentrations in hypocotyls and cotyledons were reduced in *d6pk012* and *pin347* mutants, they were comparable between the wild type, *abcb19*, as well as *kipk01* and *kipk012*, indicating that alterations of IAA content may not allow explaining *kipk01* and *kipk012* mutant phenotypes (Fig. 4G).

### Auxin accumulation patterns, but not KIPK or PIN3 polar distribution, change during gravitropic bending

The differential distribution of PIN3 during gravitropic bending between the lateral plasma membranes in hypocotyl endodermis cells had previously been reported (Rakusova et al. 2011, 2016). Accordingly, the differential cellular auxin accumulation resulting from differential PIN3 polarity would lead to a redistribution of PIN3 to terminate the bending response (Rakusova et al. 2011, 2016; Grones et al. 2018). We reasoned that defects in the above-summarized differential PIN3 distribution in the endodermis may be a cause for the observed bending defects in *kipk01* mutants.

**Figure 5.**
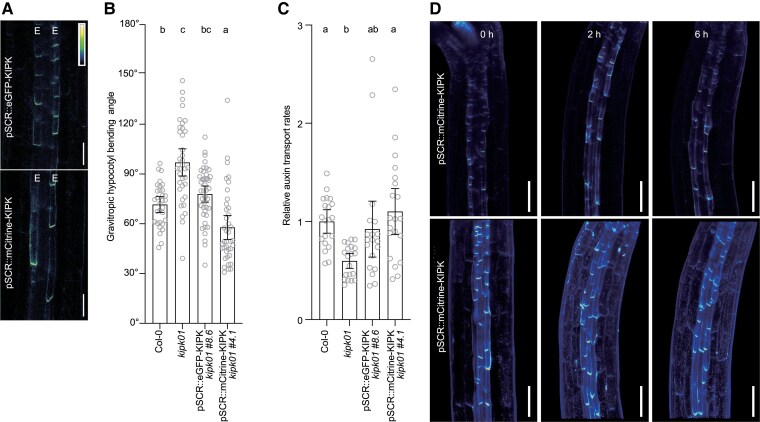
Endodermis-specific expression of *KIPK* suppresses the *kipk01* hypergravitropic responses. **A)** Representative confocal microscopy images of hypocotyls of 3-d-old dark-grown seedlings expressing pSCR::eGFP-KIPK or pSCR::mCitrine-KIPK. E, endodermal cell file. The colored bar specifies signal intensities (arbitrary units) in the confocal microscopy images. Scale bars = 50 *µ*m. **B)** Graph displaying the average and 95% confidence interval, as well as the individual results, of gravitropic hypocotyl bending experiments with 3-d-old dark-grown wild type (Col-0), *kipk01* and *kipk01* pSCR::eGFP-KIPK or *kipk01* pSCR::mCitrine-KIPK seedlings. *n* > 31 seedlings. Statistical significance was assessed using 1-way ANOVA, followed by Dunnett's T3 post hoc test for multiple comparisons. Different letters indicate significant differences between groups at *P* < 0.05. **C)** Graph displaying the average and 95% confidence interval, as well as the individual results, of relative auxin transport rates in wild type (Col-0), *kipk01* and *kipk01* pSCR::eGFP-KIPK or *kipk01* pSCR::mCitrine-KIPK seedlings. *n* = 20 × 5 seedlings. Statistical significance was assessed using 1-way ANOVA, followed by Dunnett's T3 post hoc test for multiple comparisons. Different letters indicate significant differences between groups at *P* < 0.05. **D)** Representative confocal images showing the distribution of mCitrine-KIPK before and after gravity stimulation for the times specified in the image in the central axial plane of the hypocotyl (upper images) and in the hypocotyl half cylinder (lower images; z stack). Scale bars = 100 *µ*m.

**Figure 6.**
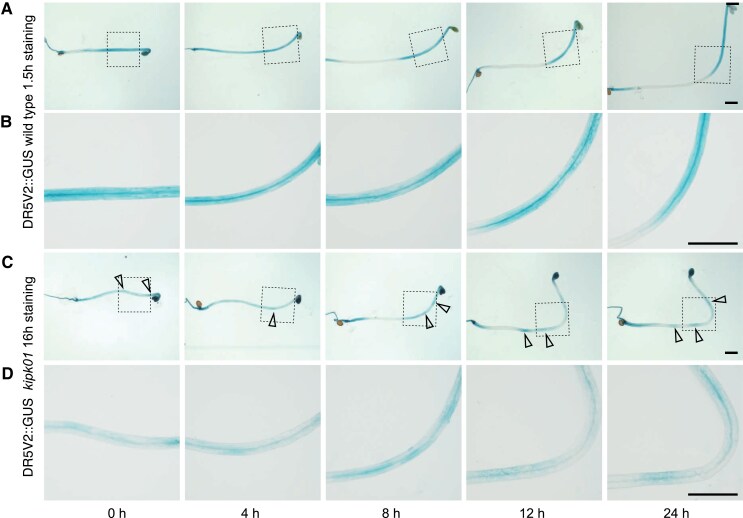
Hypocotyl overbending in *kipk01* coincides with the strong tissue level accumulation of auxin as monitored with DR5v2::GUS. **A)** and **C)** Representative photographs of 3-d-old dark-grown seedlings that had been exposed, for the times specified in the image, to a 90 °change in the gravitropic vector. Arrowheads mark the sites of auxin accumulation. Note the strong accumulation of auxin in the *kipk01* cotyledons, as well as the long staining time (16 h) required for efficient staining in the *kipk01* mutant. **B)** and **D)** Magnification from the images (squares) shown in **A)** and **C)**, respectively. Scale bars for all images of one row = 1 mm.

Our assumption that the critical response may be sensed by the endodermis found support in our observation that expression of eGFP- or mCitrine-tagged *KIPK* from the endodermis-specific *SCR* (*SCARECROW*) promoter was sufficient to rescue the bending and the auxin transport defects of *kipk01* (Fig. 5, A to C). Whereas we did not observe any changes in the cellular distribution of eGFP-KIPK or mCitrine-KIPK during gravitropic bending, we noted differences in the accumulation of auxin in *kipk01* mutants when compared to the wild type when detected with the auxin response reporter DR5v2::GUS (Fig. 5D, Fig. 6). In the wild type and in the *kipk01* mutants, local auxin accumulation, as suggested by the DR5v2::GUS reporter, coincided with the bending regions within the tissues (Fig. 6). However, whereas the wild type readily stained for the GUS reporter (1.5 hrs), staining times had to be extended for *kipk01* (16 hrs) to visualize the weak DR5v2::GUS staining in the *kipk01* hypocotyls (Fig. 6; Supplementary Fig. S7A). The latter was accompanied by a relatively strong DR5v2::GUS staining in the *kipk01* cotyledons, which again may be a reflection of the reduced auxin transport through the mutant hypocotyls (Fig. 4A, Fig. 6). We concluded that the gravitropic bending and overbending of *kipk01* mutants correlated with differential auxin accumulation in the bending regions, suggesting that auxin transport in the mutant hypocotyls may not be critically reduced to impair bending; at the same time, tissue-level auxin transport in the mutants may be misregulated, preventing feedback regulation of the bending response. The difference in the quantitative responsiveness observed with DR5V2::GUS between the wild type and *kipk01* was also observed with the auxin reporters DR5::GUS and DR5::GFP after introduction into *kipk01*, which, in view of the comparable auxin contents measured in *kipk01* and the wild type, may be indicative of the effects of the *kipk01* mutations on the cellular auxin response machinery (Fig. 4G, Fig. 6, Supplementary Fig. S7).

To be able to examine PIN3 distribution selectively in the gravity-sensing hypocotyl endodermis cells, we established lines for the expression of PIN3-GFP from the *SCR* promoter. When we measured PIN3-GFP lateral distribution by establishing the ratio between the outer (epidermis-facing) and inner (stele-facing) lateral membranes in lower, as well as the upper endodermal cells, we did not detect differential PIN3-GFP distribution during the gravitropic response, a finding that is in conflict with previously reported findings (Supplementary Fig. S8, A and B; Rakusova et al. 2011, 2016; Grones et al. 2018). From these data, we concluded that, at least in our experimental conditions, a lateral redistribution of PIN3 cannot explain gravitropic hypocotyl bending in the wild type or the overbending defect of the mutants. We further employed an anti-GFP antibody to monitor the distribution of pPIN3::PIN3-GFP between the wild type and the *kipk01* mutant. However, these experiments did not reveal any changes in the abundance of PIN3-GFP or in the abundance of a phosphorylated PIN3-GFP form that can be separated on the respective immunoblots between the wild type and the *kipk01* mutants (Supplementary Fig. S8C; Willige et al. 2013). We concluded that dynamic changes in the distribution of PIN3-GFP, its abundance, or phosphorylation cannot be correlated with the exaggerated hypocotyl bending phenotype of *kipk01* mutants.

### *ZWI* and PERK8, *PERK9,* and *PERK10* are not required for gravitropic hypocotyl bending

KIPKs had previously been reported to interact with KINESIN-LIKE CALMODULIN-BINDING PROTEIN (KCBP), which is defective in the trichome formation mutant *zwichel* (*zwi*; Oppenheimer et al. 1997; Day et al. 2000). When we examined hypocotyl gravitropism in the *zwi* mutant, we did, however, not observe negative gravitropic bending defects in *zwi* mutants (Supplementary Fig. S9, A and B). Similarly, did we not observe a corresponding phenotype in mutants of the previously identified KIPK interactors of the PERK family when examining *perk8 perk9 perk10* triple mutants (Supplementary Fig. S9, C and D; Humphrey et al. 2015). We therefore concluded that these 2 interactors and their homologs may not act together with KIPK and KIPKL1 in the context of gravitropic hypocotyl bending.

### *KIPK* and *KIPKL1* are required for efficient seedling penetration through the soil

**Figure 7.**
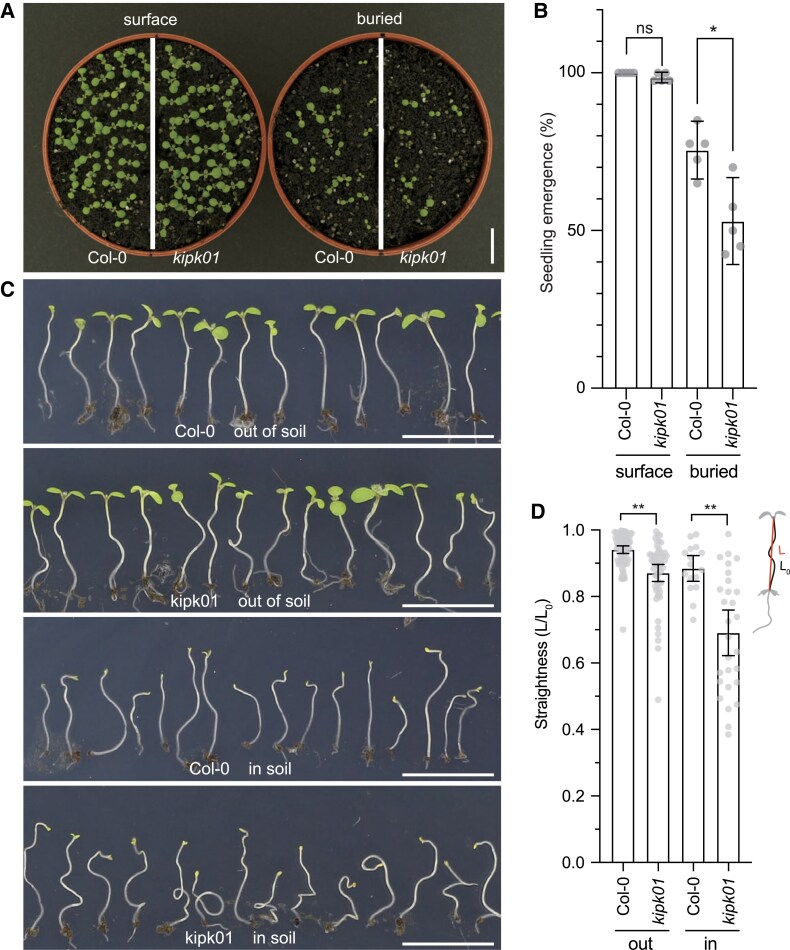
*KIPK* and *KIPKL1* are required for efficient soil penetration of seedling hypocotyls. **A)** Top view of wild-type (Col0) and *kipk kipkl1* mutant seedlings sawn on the surface of soil or buried 1 cm below the soil. Scale bar = 1 cm. **B)** Graph displaying the average and 95% confidence interval, as well as the individual data points from experiments with seeds sown on the soil surface or buried in the soil. *n* = 5 × 40 seedlings. **C)** Photographs of seedlings germinated in the soil after emergence from the soil (out of soil) or without emergence from the soil (in soil). Scale bar = 1 cm. **D)** Graph displaying the average and 95% confidence interval, as well as the individual data points from straightness measurements of seedlings germinated in the soil after emergence from the soil (out) or without emergence from the soil (in), as determined by division of the lengths (L_0_) and the heights (L) of the seedling hypocotyls. *n* > 15 seedlings. Welch's *t*-test **B, D)**. *P < 0.05; **P < 0.01; ns, not significant.

Negative gravitropic growth of elongating hypocotyls is essential for soil penetration during etiolated seedling growth after seed germination. However, elongating hypocotyls constantly need to adjust their growth direction in the soil to efficiently grow around obstacles and resume negative gravitropic growth with the limited resources of the seed to efficiently reach the sunlight for autotrophic growth. We reasoned that KIPK and KIPKL1 may be essential for the efficient soil penetration of germinated seedlings. To test this hypothesis, we buried wild-type and *kipk01* mutant seeds 1 cm under the soil surface and, as a control, germinated the seedlings on the soil surface. When germinated on the soil surface, almost all seeds germinated (Fig. 7, A and B). When buried in the soil, approximately 75% of the wild-type seedlings emerged from the soil, but this number was reduced to 50% in the case of *kipk01* mutants (Fig. 7, A and B). In both populations, in *kipk01* seedlings that had emerged from the soil and those that had not emerged from the soil, we observed enhanced hypocotyl bending and curving, which we quantified by determining seedling straightness (Fig. 7, C and D). Naturally, these phenotypes were more pronounced in seedlings that had not penetrated from the soil, but straightness was reduced in *kipk01* when compared to the wild type (Fig. 7, C and D). We concluded that KIPK and KIPKL1 are required for re-establishing proper negative gravitropic growth of seedling hypocotyls after their reorientation forced by contact with soil particles during soil penetration.

## Discussion

In the present study, we identify KIPK and KIPKL1 as redundant regulators of proper hypocotyl bending during negative gravitropic responses. The overbending phenotypes can be observed in *kipk kipkl1* mutants after being exposed to a change in the gravitropic vector, as well as in *kipk kipkl1* mutants growing through soil where the soil particles represent growth-restricting obstacles that mechanically induce changes in the gravity vector. Our work identifies auxin transport regulation as a possible mechanism of how KIPK and KIPKL1 prevent hypocotyl overbending in the wild type.

KIPK and KIPKL1 are polarly localized plasma membrane-associated proteins whose membrane association is impaired in the presence of PAO (phenylarsine oxide), an inhibitor of phosphoinositol-4-phosphate synthesis (Simon et al. 2016). Although it is therefore very likely that the interactions at the plasma membrane, e.g. with phosphoinositol-4-phosphate or its derivatives, are mediated by interactions with positively charged basic amino acids in KIPK and KIPKL1, the mutation of a strongly polybasic motif with 12 basic amino acids found in the KIPK N-terminus did not abolish its plasma membrane association and did not impair the biological functionality of the protein in complementation experiments. In recent work, we had shown that the AGC1 kinase D6PK interacts with the plasma membrane through S-acylation of cysteines at conserved CXX(X)P motifs (Graf et al. 2024). KIPK and KIPKL proteins also contain such CXX(X)P motifs, and S-acylation is likely an additional membrane-anchoring process (Bassukas et al. 2022). A further mechanism for membrane attachment of the KIPK and KIPKL proteins may be through interactions with other membrane-bound proteins.

Although KIPKL2, the third family member, is homologous to KIPK and KIPKL1 and shares high levels of sequence similarity, along with the pronounced polar distribution at the basal plasma membrane with KIPK and KIPKL1, our data clearly indicate that KIPKL2 has distinct functions. First, expression of *KIPKL2*, unlike the expression of *KIPK* or *KIPKL1* from a *KIPK* promoter fragment, cannot suppress the phenotype of the *kipk012* mutant. Second, the loss of *KIPKL2* in the *kipk012* triple mutant does not quantitatively enhance the phenotype of the *kipk01* double mutant. Third, in auxin transport assays, KIPKL2 is less efficient than KIPK and KIPKL1 in activating the auxin transporter PIN3. Then, while KIPKL2 shares the strong polar distribution at the basal plasma membrane with KIPK and KIPKL1 and their affinity for phospholipids, KIPKL2 differs from the other 2 family members with regard to the presence of a polybasic motif in the middle domain of KIPKL2, which is reminiscent of similar polybasic motifs in the middle domains of D6PK, D6PK-LIKE proteins or PAX but absent from KIPK or KIPKL1 (Barbosa et al. 2016; Bassukas et al. 2022). We conclude from these observations that KIPKL2 is neither biochemically nor biologically redundant with KIPK and KIPKL1. With regard to the latter point, we noted with interest that the promoter activity of *KIPKL2* in the meristematic zone of the root tip is suggestive for a cell cycle-dependent regulation. Further research is needed to elucidate the function of KIPKL2 and its functional distinction from KIPK and KIPKL1 at the biological, biochemical, and cell biological level.

Like D6PK, D6PKL and PAX kinases, KIPK and KIPKL1 are polarly localized at the basal (rootward) plasma membrane, where they colocalize with polarly localized PINs (Barbosa et al. 2014; Weller et al. 2017; Marhava et al. 2018). KIPK and KIPKL1 further phosphorylate “canonical” PIN transporters in vitro. The requirement of PINs and auxin transport for the initiation of tropic bending responses prevented us from examining their role during the termination of the bending response, which is impaired in the *kipk kipkl1* mutants, or bending during hypocotyl elongation growth in the soil. Since we measured reduced auxin transport in *kipk kipkl1* mutant hypocotyls while auxin levels remained similar between the wild type and the *kipk kipkl1* mutants, we argue that a reduction of KIPK and KIPKL1-dependent PIN-mediated polar auxin transport, at least in part, contributes to the growth defects of *kipk kipkl1* mutants. Based on the observation that all 3 auxin reporters introduced into *kipk kipkl1*, DR5V2::GUS, DR5::GUS, and DR5::GFP, show reduced activity in the mutant, we also need to consider that reduced cellular auxin responses or reduced coordination between cellular auxin responses and auxin transport at least contribute to the phenotype.

Previous work on hypocotyl gravitropic bending had suggested that the bending process was accompanied by a redistribution of the auxin transporter PIN3 in gravity-sensing endodermis cells, in line with the assumption that a relative increase in auxin distribution at the lower hypocotyl cell files would promote the cell elongation required for bending (Rakusova et al. 2011). Further, it had been postulated that the consequent auxin accumulation at the lower hypocotyl cell files would induce a second lateral redistribution of PIN3 to terminate the bending response (Rakusova et al. 2016; Grones et al. 2018). We reasoned that differences in this PIN3 distribution may be causal for the bending phenotypes observed in *kipk01* mutants. However, when we examined the distribution of PIN3-YFP expressed from the endodermis-specific *SCR* promoter in cells undergoing differential elongation, we failed to observe the previously reported PIN3 redistributions between the 2 lateral membranes. Our observation that there is no dynamic redistribution of PIN3 between the lateral plasma membranes during gravitropic responses is in conflict with the previously published work that described such a redistribution and also the termination of the bending response as a consequence of cellular auxin accumulation (Rakusova et al. 2011, 2016; Grones et al. 2018). We therefore suggest that the previously established model of an auxin-regulated differential lateral PIN3 distribution during negative hypocotyl bending, although attractive, may require a further detailed re-examination. The absence of a PIN3-YFP redistribution also prevented us from using this cell biological readout for the characterization of the overbending phenotype of *kipk kipkl1* mutants.

Another possible mechanism for the regulation of gravitropic hypocotyl bending through the polarly localized KIPK and KIPKL1 kinases would have been a cellular redistribution of the KIPK and KIPKL1 proteins during gravitropic response. However, similarly to our inability of observing such polarity changes with PIN3, we also did not observe changes in the polar distribution of KIPK or KIPKL1 during gravitropic responses when examined in the endodermis, a cell file where *KIPK* expression from the *SCR* was sufficient to suppress the *kipk01* phenotype. Dynamic kinase polarity changes may thus also not be at the basis of the observed phenotypes.

Based on phenotypical similarities, we also considered a functional interplay between KIPK, KIPKL1, and ABC family auxin transporters. We found, however, that the mutant of the *ABCB19* transporter had different defects in auxin transport than *kipk kipkl1* mutants with regard to the defects in auxin transport observed in *kipk kipkl1* mutants and their sensitivity to the inhibitor NPA. We therefore considered a functional relationship between the kinases and the ABCB transporters unlikely. Recent work showed that brassinosteroids, not auxin, are the primary cargoes of the ABCB transporters (Ying et al. 2024). This and other previously published work suggest that ABC transporters may have a broader range of substrates, which we did not analyze in our work (Blakeslee et al. 2007; Yang and Murphy 2009). Therefore, we can also not fully exclude the possibility that the KIPK and KIPKL kinases act in the ABCB19 pathway.

KIPK had previously been reported to be an interactor of the kinesin ZWI (Day et al. 2000). Although *kipk kipkl1* did not display reductions in trichome branch numbers as reported for *zwi* and, inversely, *zwi* mutants did not display the overbending phenotype of *kipk kipkl1* mutants, there may be, as yet unknown, biological processes where a ZWI kinesin regulation by KIPK/KIPKL kinases may be biologically relevant. Similarly, we did not observe hypocotyl bending phenotypes in mutants of the reported KIPK-interacting *PERK* kinases (Humphrey et al. 2015). PERK kinases are cell surface proteins with extension motifs and cytoplasmic kinase domains (Borassi et al. 2016). PERKs have a proposed role in cell wall integrity sensing that link cell wall interactions with cytoplasmic signaling (Borassi et al. 2016). In line with the function proposed for KIPK and KIPKL1, gravitropic bending involves cell expansion, requiring cell wall loosening, deposition of new cell wall materials, and subsequent cell wall rigidification and, thus, could require proteins with the proposed functionality of PERKs. Thus, the reported interaction between KIPK, KIPKL1 and PERKs may deserve further research and could reveal biological functions beyond the reported role of PERKs in root growth on media containing high sucrose levels (Humphrey et al. 2015).

In conclusion, our research reveals a hitherto unknown biological function of the KIPK and KIPKL1 proteins in controlling gravitropic hypocotyl bending, which is also biologically significant when seedling hypocotyls are penetrating the soil. Even though it is very likely that KIPK and KIPKL1, like other AGC1 kinases, regulate auxin transport through PIN protein phosphorylation, the initiation of gravitropic bending is unaffected by the decreased auxin transport in *kipk kipkl1* mutants or the decreased auxin response in the mutant hypocotyls. Instead, it seems that KIPK and KIPKL1 are a part of a feedback process that terminates gravitropic bending. PIN control and dynamic auxin transport may be part of this suggested feedback mechanism.

## Materials and methods

### Biological material

All experiments were performed with *A. thaliana* Columbia-0 (Col-0) as the wild-type control. The insertion alleles *kipk-1* (SALK_065651C), *kipkl1-1* (GABI_175F10), *kipkl1-2* (SALK_200701C), *kipkl2-1* (SALK_087379C), and *kipkl2-2* (SALK_015563) were obtained from the Nottingham Arabidopsis Stock Center (NASC, Nottingham, UK) and genotyped for homozygosity before use for experimental work. The alleles *kipk-1*, *kipkl1-1*, and *kipkl2-1* were used to generate *kipk01*, kipk02, and *kipk12* double and *kipk012* triple mutants. *kipk-1* and *kipkl1-2* were used to generate the *kipk01-2* double mutant.

The following mutants, alleles, and transgenes have been described elsewhere: *abcb19* (SALK_033455; Lewis et al. 2007); 35S::YFP-D6PK (Zourelidou et al. 2009); *d6pk d6pkl1 d6pkl2* (*d6pk012*; Zourelidou et al. 2009); *pin3-3* (2 bp deletion; Friml et al. 2002); *pin3-3 pin4-101* (GABI_593F01) *pin7-102* (SALK_062056; *pin347*; Willige et al. 2013); pSCR::PIN3-YFP (Rakusova et al. 2011); pPIN3::PIN3-GFP (Zadnikova et al. 2010); *zwi* (SALK_031704; Humphrey et al. 2015); *perk8-1* (SALK_129961) *perk9-1* (SALK_014687) *perk10-1* (SALK_022872; Humphrey et al. 2015).

Primers used for genotyping are listed in Supplementary Table S1.

### Plant cultivation

For growth in sterile culture, seeds were surface-sterilized for 7 min in 25% DanKlorix hygiene cleaner (CP GABA, Hamburg, Germany) and 0.05% Triton X-100, followed by 5 washes with sterile water. Sterilized seeds were kept in the dark at 4 °C for 2 to 4 d for stratification and germinated on ½ Murashige and Skoog (½ MS) medium (Duchefa, Harlem, The Netherlands) supplemented with 0.05% 2-(N-morpholino) ethanesulfonic acid, 1% sucrose, and 0.6% or 0.8% agar, depending on whether the seeds were grown on horizontally (0.6%) or vertically (0.8%) oriented plates. Seedlings on plates were grown at 21 °C in the dark or in continuous light (110 *µ*M m^−2^ s^−1^). For growth on soil, plants were grown at 21 °C with a 16 h photoperiod (120 *µ*M m^−2^ s^−1^) and 65% relative humidity.

### Cloning procedures and transgenic lines

pGreenII0229-pKIPK::YFP-KIPK (pKIPK::YFP-KIPK), expressing a genomic variant of *KIPK*, was used for phenotype complementation. To obtain pGreenII0229-pKIPK::YFP-KIPK, a genomic fragment of *KIPK* was introduced into pDONR201 and subcloned into pGreenII0229-pD6PK::YFP-GW using Gateway methodology to obtain pGreenII0229-pD6PK::YFP-KIPK (Invitrogen, Carlsbad, CA, USA). Subsequently, the *D6PK* promoter fragment was replaced by a 2049 bp *KIPK* promoter PCR fragment flanked by *KpnI* and *XhoI* sites in pGreenII0229-pD6PK::YFP-KIPK.

To generate constructs for *KIPK* or *KIPKL* overexpression, their full-length coding sequences were PCR-amplified from Col-0 cDNA and cloned into pDONR201 using BP Clonase (Invitrogen, Carlsbad, CA, USA) and subsequently subcloned into pUBN-Dest-eGFP-GW (Grefen et al. 2010) to obtain pUBN::eGFP-KIPK (eGFP-KIPK), pUBN::eGFP-KIPKL1 (eGFP-KIPKL1) and pUBN::eGFP-KIPKL2 (eGFP-KIPKL2). The *UBN10* promoter fragment between the *EcoRI* and *XhoI* sites was then replaced by *KIPK* (2018bp) or *KIPKL1* (2142 bp) native promoter PCR fragments to obtain, using standard cloning procedures, pKIPK::eGFP-KIPK and pKIPKL1::eGFP-KIPKL1 for mutant complementation. *KIPKL2*, *D6PK,* and *KIPK* mutant variants were introduced by Gateway LR cloning (Invitrogen, Carlsbad, CA, USA) into pKIPK::eGFP-GW, which had been obtained with a BP Clonase (Invitrogen, Carlsbad, CA, USA) reaction between pKIPK::eGFP-KIPK and pDONR201, to obtain pKIPK::eGFP-KIPKL2, pKIPK::eGFP-D6PK and pKIPK::eGFP-KIPK mutant variants. pSCR::eGFP-KIPK was subsequently generated by replacing the fragment between the *EcoRI* and *XhoI* sites with the 2049bp *SCARECROW* (*SCR*) promoter PCR fragment.

For endodermis-specific expression of *KIPK*, the 2049bp *SCR* promoter fragment and a *BsaI* site-cured *KIPK* coding sequence were ligated into pGGA000 and pGGC000 entry vectors, respectively, to subsequently generate pFASTR-pSCR::mCitrine-KIPK (pSCR::mCitrine-KIPK) in pFASTR-AG by Greengate cloning (Lampropoulos et al. 2013; Decaestecker et al. 2019).

Point mutations in *KIPK* were introduced by site-directed mutagenesis into pDONR201-KIPK as a template to obtain the KR12A variant with mutations of 12 basic amino acids defining the domain with a high (>0.6) BH score located between R332 and K355 (R332A, K333A, R336A, R340A; K342A, K345A, K346A, K347A, K351A, K352A, K353A, K355A; Fisher and Pei 1997).

To generate pKIPK::eGFP-GUS, as well as pKIPKL::eGFP-GUS vectors, *KIPK* (2049bp), *KIPKL1* (2102 bp), and *KIPKL2* (2313 bp) promoter fragments were amplified from Col-0 genomic DNA, cloned into pDONR201, and subsequently subcloned into pFASTG04 using Gateway technology (Invitrogen, Carlsbad, CA, USA; Shimada et al. 2010).

All constructs for the expression of *KIPK/KIPKL* or mutant variants under the control of the *pKIPK*, *pKIPKL1*, *pKIPKL2*, or *pSCR* promoters were transformed into the *kipk01* mutant to analyze mutant complementation and protein localization. pKIPK/pKIPKL::eGFP-GUS constructs were transformed into Col-0 for gene expression pattern visualization. For each construct, homozygous lines of at least 3 independent lines were analyzed.

To obtain modified DR5v2::GUSm, the previously published DR5v2::GUS vector (Hayes et al. 2019), lacking a terminator sequence for *ß-glucuronidase* (*GUS*) expression, was modified by introducing a 256 bp terminator fragment of *HEAT SHOCK PROTEIN 18.2* into the *SacI* site. DR5v2::GUSm was transformed into *kipk01* and backcrossed to the Col-0 wild type to analyze auxin distribution.

The coding sequences of *KIPK*, KIPKL1, and *KIPKL2* were ligated into the blunt-ended pOO2 vector, obtained by *Eco32*I, *Sma*I, and *Eco32*I restriction digestion, respectively.

All the PCR amplification for cloning were carried out with PrimeSTAR Max DNA Polymerase (Takara, Kyoto, Japan). All primers used for cloning and mutagenesis are listed in Supplementary Table S1.

### Protein alignment

Full-length *A. thaliana* AGC1 and AGC4 protein sequences were used for protein alignment with the ClustalW algorithm of the Geneious software (Thompson et al. 1994). Phylogenetic analysis was done using the MEGA-X software with the Neighbour-Joining method (Saitou and Nei 1987).

### RT-PCR

Total RNA was extracted from 5-d-old light-grown seedlings using the NucleoSpin RNA Plant kit (Macherey Nagel, Düren, Germany). cDNA was synthesized with a RevertAid First Strand cDNA synthesis kit (Thermo Scientific, Waltham, MA, USA), and the *KIPK/KIPKL* open reading frames were amplified with DreamTaq DNA Polymerase (40 cycles; Thermo Scientific, Waltham, MA, USA). ACTIN2 was analysed as a control gene (20 cycles). Primers are listed in Supplementary Table S1.

### Tropism and soil growth assays

For hypocotyl negative gravitropism assays, seeds were germinated for 18 to 24 h in the light and then grown for 2.5 to 3 d in the dark on vertically oriented plates covered in multiple layers of aluminum foil. The hypocotyls of the etiolated seedlings were straightened in the direction of the gravity vector in safe green light and allowed to recover for 1 to 2 h. For negative hypocotyl gravitropism assays, the plates were turned by 90° for gravity stimulation, and images were obtained after 24 h or, for the time-resolved analysis, after shorter time intervals as specified.

For phototropism assays, seedlings were prepared in an identical manner, and plates were then transferred to a FloraLED chamber (CLF Plant Climatics, Wertingen, Germany) and illuminated with 1 μmol m^−2^ s^−1^ blue light from a 90° angle for 16 h. Hypocotyls were photographed with a Canon EOS 650D camera (Canon, Tokyo, Japan) and hypocotyl bending was determined with ImageJ (Fiji; https://imagej.net/ij/).

Root positive gravitropism was measured from 6-d-old light-grown seedlings that had been aligned in the direction of the gravity vector on vertically oriented plates. Roots were photographed 8 h after reorienting the plates by 90° and root bending was determined with ImageJ (Fiji; https://imagej.net/ij/).

For shoot negative gravitropism assays, 46-d-old *A. thaliana* plants with primary inflorescence lengths of ca. 10 cm were reoriented by 90° and allowed to grow for 1 or 22 h, as specified, in 2 independent experiments. Plants were photographed with a Canon EOS 750D camera (Canon, Tokyo, Japan). Shoot angles were measured using ImageJ (Fiji; https://imagej.net/ij/). GraphPad Prism 9 (GraphPad Software, San Diego, CA, USA) and Origin 8.0 (OriginLab, Northhampton, MA, USA) were used for statistical analyses and plotting.

To determine seedling growth in soil, seeds were sown on 1/2 mS agar plates and exposed to light for 6 h. The absolute seed number was determined, and seeds were transferred to the soil surface or covered under 1 cm of loamy soil, prepared by blending sieved organic matter with sand (average diameter 1 mm) in a 1:5 ratio based on dry weight. Seed germination and seedling emergence were assessed after growth at 22 °C for 7 d.

### GUS staining

To determine *KIPK/KIPKL* expression from GUS-GFP reporter lines or auxin distribution from DR5v2::GUS, 3-d-old dark-grown seedlings or 3- and 5-d-old light-grown seedlings were fixed in 90% acetone at −20 °C for 2 h, washed twice with water, and then placed into GUS staining buffer (10 mm EDTA, 50 mm sodium phosphate [pH 7.0], 0.1% [v/v] Triton X-100, 0.5 mm K_3_Fe(CN)_6_, 0.5 mm K_4_Fe(CN)_6_, 1 mg/mL X-GlcA). Samples were placed under vacuum for 30 min to improve penetration of the staining solution. proKIPK/KIPKL::GUS-GFP, DR5v2::GUS, and DR5v2::GUS *kipk01* were incubated at 37 °C for 18 or 1.5 and 16 h, respectively. All samples were cleared in 70% (v/v) ethanol and imaged with a Leica MZ16 (Leica, Wetzlar, Germany) or Zeiss AXIO Zoom.V16 (Zeiss Oberkochen, Germany) microscope equipped with a Leica DMC5400 or Axiocam 305 color camera system, respectively.

### Confocal microscopy

To visualize mCitrine/YFP-KIPK/D6PK and eGFP-KIPK/KIPKL fusions, seedlings were cleared with ClearSee or ClearSeeAlpha for 2 to 4 d (Kurihara et al. 2015, 2021). DR5::GFP, pSCR::PIN3-YFP and pPIN3::PIN3-GFP were directly detected in 3-d-old dark-grown seedlings at the specified time points with an Olympus FV1000 confocal microscope with a high-sensitivity detector unit using a 515 nm laser and a 520 to 550 nm band pass filter for mCitrine/YFP detection and a 488 nm laser and a 505 to 540 nm band pass filter for eGFP detection and 20 × and 40 × objectives (Olympus, Tokyo, Japan). Laser intensity was set to 30% of the maximum output for D6PK and KIPK/Ls due to their low expression levels. For PIN3-GFP/YFP and DR5::GFP, laser intensity was reduced to around 10% with an appropriate gain to avoid signal saturation. Laser intensity and gain were kept constant for each sample in one experiment to ensure uniform imaging conditions. The mean intensities of PIN3-YFP on the inner and outer sides of the endodermis were measured with ImageJ (Fiji) by drawing freehand lines along the entire lateral plasma membrane at the 2 lateral sides of the cell (https://imagej.net/ij/).

### Chemical inhibitor experiments

For chemical inhibitor treatments, roots of 5-d-old light-grown seedlings expressing YFP-KIPK and YFP-D6PK were imaged; the respective concentration and treatment duration for each chemical is indicated in figure legends as previously described (Gao et al. 2013; Barbosa et al. 2016). Taxol (Sigma, Taufkirchen, Germany) was directly added into ½ MS medium at the specified concentrations; seedlings were grown for 4 d in the dark on vertically oriented plates covered in multiple layers of aluminum foil prior to imaging and hypocotyl length measurements with ImageJ (Fiji; https://imagej.net/ij/).

### PIP strip experiment

For recombinant protein expression and purification, GST-KIPK and GST-KIPKL proteins were expressed in *Escherichia coli* Rosetta (DE3). Free GST protein was expressed from a pGEX-KG empty vector as a negative control (ATCC, Manassas, VA, USA; Guan and Dixon 1991). All GST-tag proteins were purified using Protino Glutathione Agarose 4B (Macherey-Nagel, Düren, Germany). Lipid-binding assays were performed following the previous description using TBS buffer on PIP strips P-6001, as described (Echelon, San José, CA, USA; Barbosa et al. 2016).

### In vitro phosphorylation experiments

For in vitro phosphorylation reactions, MBP fusion proteins were expressed in *E. coli* Rosetta (DE3) pLysS (Novagen, Darmstadt, Germany) and purified using Amylose Resin (New England Biolabs, Ipswich, MA, USA). 0.5–1 *μ*g purified GST-PIN3 cytoplasmic loop (CL) and 0.1–0.2 µg recombinant kinase were used for each reaction in 1 × kinase buffer (25 mm Tris pH 7.5, 5 mm MgCl_2_, 1 mm DTT), 100 *μ*M ATP and 3 μCi [γ-^32^P] ATP [10 μCi μL^−1^ stock] (Hartmann Analytic, Braunschweig, Germany). Reactions were incubated at 30 °C for 1 h and stopped by the addition of 5 × Laemmli SDS loading buffer and boiling at 56 °C for 10 min. Reactions were subsequently separated on a 4% to 12% iD PAGE Gel (Eurogentec, Liege, Belgium). The gels were washed twice with 5% trichloroacetic acid, stained with Coomassie Brilliant Blue (CBB), destained with destaining buffer (40% ethanol, 7% acetic acid), and dried overnight. Autoradiography was performed for 6 h (MBP-KIPK) or 5 d (MPB-KIPKL1 and MBP-KIPKL2) at −80 °C on X-ray film (CEA, Cadarache, France).

### In vivo auxin transport assay

Auxin transport assays in hypocotyls of etiolated seedlings were carried out as previously reported, with slight modifications (Willige et al. 2013). Briefly, ^3^H-IAA (25 Ci mmol^−1^; RC Tritec, Teufen, Switzerland) was dissolved to a final concentration of 400 nm in 5 mm MES (pH 5.5), 1% glycerol. Four-d-old dark-grown seedlings with comparable lengths were transferred onto Parafilm (Beemis Company, Neenah, WI, USA) strips placed on the surface of ½ MS vertical plates. Seedlings were aligned on a 6 mm Parafilm strip with the cotyledons and the apical part of the hypocotyl covering 5 mm of the Parafilm strip, allowing for the application of 0.5 μL ^3^H-IAA solution to the cotyledons of each seedling. Seedlings were then incubated vertically in the dark for 4 h. Subsequently, the lower part of the hypocotyl was excised. For scintillation counting, 5 seedlings were grouped into one scintillation vial containing 2 mL Ultima Gold liquid scintillation cocktail and counted in a Tri-Carb 4910TR Liquid Scintillation counter (Perkin Elmer, Rodgau, Germany). The results from all auxin transport measurements are provided in Supplementary Table S2.

### Oocyte auxin efflux assay

For auxin transport assays conducted in oocytes, *Xenopus laevis* oocytes were collected as previously described (Zourelidou et al. 2014; Fastner et al. 2017). cRNA was synthesized using the mMessage Machine SP6 Kit (Life Technologies, Carlsbad, CA, USA), and cRNA concentration was adjusted to 300 ng/µL PIN and 150 ng/µL protein kinase, respectively. Oocytes were injected the day after surgery with ∼50 nL of a 1:1 mixture of cRNAs for PIN and the respective protein kinase. If only PIN or protein kinase cRNA was injected, the cRNA was mixed 1:1 with water (mock control). Following injection, oocytes were incubated in Barth's solution containing 88 mm NaCl, 1 mm KCl, 0.8 mm MgSO_4_, 0.4 mm CaCl_2_, 0.3 mm Ca(NO_3_)_2_, 2.4 mm NaHCO_3_, 10 mm HEPES (pH 7.4) supplemented with 50 *µ*L gentamycine at 16 °C for 4 d to allow for protein synthesis. An outside medium buffer at pH 7.4 was chosen to minimize passive rediffusion of IAA into the oocytes, which increases at acidic pH. At the beginning of the experiment, 10 oocytes per time point were injected with 50 nL of a 1:5 dilution (in Barth's solution) of [^3^H]-IAA, 25 Ci/mmol; 1 mCi/mL (ARC, St. Louis, MO, USA or Tritec, Teufen, Switzerland) to reach an intracellular oocyte concentration of ∼1 *µ*M [^3^H]-IAA based on an estimated oocyte volume of 400 nL (Broer 2010). After [^3^H]-IAA injection, oocytes were placed in ice-cold Barth's solution for 2 min to allow substrate diffusion and closure of the injection spot. Subsequently, oocytes were washed and transferred to Barth's solution at 21 °C to allow for auxin efflux. To stop auxin efflux, oocytes were washed twice and lysed individually in 100 µL 10% SDS (w/v) at selected time points and the residual amount of [^3^H]-IAA in each oocyte was determined by liquid scintillation counting. At least 7 oocytes were measured per time point and mock as well as other negative controls were performed with the same oocyte batch to account for differences between batches. The relative transport rates of an experiment were determined by linear regression. Transport rates of different biological replicates, i.e. oocytes collected from different donor animals were averaged and are presented as mean and standard error of at least 3 biological replicates.

### Auxin measurements

IAA measurements were performed from hypocotyls and cotyledons of 4-d-old dark-grown seedlings as previously described (Simura et al. 2018).

### Immunoblot analyses

Three-day-old dark-grown seedlings expressing pPIN3::PIN3-GFP in Col-0 and *kipk01* backgrounds were turned 90° and kept for 2 h as gravity-stimulated samples. Gravity-stimulated seedlings and non-stimulated control seedlings were ground in liquid nitrogen and homogenized in extraction buffer (50 mm Tris-HCl [pH 7.5], 150 mm NaCl, 0.2% Triton X-100, 100 *μ*M MG132, 1 mm PMSF, 1 × cOmplete Protease Inhibitor Cocktail (Roche, Penzberg, Germany) supplemented with 1 × PhosSTOP (Roche, Penzberg, Germany). Extracts were cleared by centrifugation at 5,000 *g* for 5 min. The supernatant was incubated with GFP-Trap Magnetic Agarose (Chromotek, Planegg, Germany) at 4 °C for 2 h. The beads were washed 3 times with extraction buffer. A total of 400 U Lambda Protein Phosphatase (New England Biolabs, Frankfurt, Germany) in NEBuffer, containing 10 mm MnCl_2_, and purified GST-KIPK in kinase buffer (25 mm Tris pH 7.5, 5 mm MgCl_2_, 1 mm DTT, 1 mm ATP) were added to 2 of the PIN3-GFP immunoprecipitates, respectively. Dephosphorylation and phosphorylation reactions were both incubated at 30 °C for 1 h. All protein samples were denatured at 42 °C for 10 min after adding 5 × Laemmli buffer. An anti-GFP (laboratory stock) and an anti-rabbit HRP-conjugated secondary antibody (Sigma, Taufkirchen, Germany) were used for western blot detection. Chemiluminescence was generated with SuperSignal West Femto Maximum Sensitivity Substrate (Thermo Scientific, 34096) and detected with a Fujifilm LAS 4000 mini (Fuji, Tokyo, Japan). Coomassie Brilliant Blue-stained gels of the lysate after homogenization were used as loading controls.

### Statistical analysis

Information on the statistical analyses is provided in the respective figure legends and in Supplementary Table S3. For normally distributed data with unequal variances between groups, statistical analyses were performed using Welch's *t*-test or 1-way ANOVA (Brown–Forsythe and Welch ANOVA tests) followed by Dunnett's T3 multiple comparison post hoc test. For normally distributed data and equal variance between groups, statistical analyses were performed with Student's *t*-test or ordinary one-way ANOVA followed by Tukey's post hoc test. All analyses were conducted using GraphPad Prism 9.

### Accession numbers

ABCB19 (AT3G28860), D6PK (AT5G55910), D6PKL1 (AT4G26610), D6PKL2 (AT5G47750), D6PKL3 (AT3G27580), KIPK (AT3G52890), KIPKL1 (AT2G36350), KIPKL2 (AT5G03640), PERK8 (AT5G38560), PERK9 (AT1G68690), PERK10 (AT1G26150), PID (AT2G34650), PIN3 (AT1G70940), PIN4 (AT2G01420), PIN7 (AT1G23080), SCR (AT3G54220), ZWI/KCBP (AT5G65930).

## Supplementary Material

koaf056_Supplementary_Data

## Data Availability

Unless the data underlying this article are available in the article and in its online supplementary material, the original data will be shared on reasonable request to the corresponding author.

## Supplementary data

The following materials are available in the online version of this article.

**Supplementary Figure S1.** Schematic representation of KIPK and KIPKL proteins in the context of *Arabidopsis thaliana* AGC1 and phototropin AGC4 kinases.

**Supplementary Figure S2.** Expression analyses using *promoter::GUS* fusions reveal a broad expression pattern of the *KIPK* and *KIPKL* genes.

**Supplementary Figure S3.** KIPK, KIPKL1 and KIPKL2 bind anionic phospholipids.

**Supplementary Figure S4.** A polybasic region in the N-terminus of KIPK is dispensable for plasma membrane interactions.

**Supplementary Figure S5.** Mutants of *KIPK* and *KIPKLs* do not display apparent growth defects.

**Supplementary Figure S6.**  *kipk01* mutants are defective in bending during the later stages of the bending response.

**Supplementary Figure S7.**  *kipk01* mutants display reduced auxin responses.

**Supplementary Figure S8.** PIN3-GFP lateral distribution is stable during the gravitropism response.

**Supplementary Figure S9.**  *ZWI*, *PERK8*, *PERK9*, and *PERK10* are not required for gravitropic hypocotyl bending.

**Supplementary Table S1.** Primers used in this study.

**Supplementary Table S2.** Results from auxin transport measurements.

**Supplementary Table S3.** Results from statistical analyses.

## Dive Curated Terms

The following phenotypic, genotypic, and functional terms are of significance to the work described in this paper:

zwi Gramene: AT5G65930
zwi Araport: AT5G65930

WAG2 Gramene: AT3G14370
WAG2 Araport: AT3G14370

CLF Gramene: AT2G23380
CLF Araport: AT2G23380

LAS Gramene: AT1G55580
LAS Araport: AT1G55580

WAG1 Gramene: AT1G53700
WAG1 Araport: AT1G53700

ABCB19 Gramene: AT3G28860
ABCB19 Araport: AT3G28860

PIN3 Gramene: AT1G70940
PIN3 Araport: AT1G70940

BFA Gramene: Brefeldin A
BFA Araport: Brefeldin A

## References

[koaf056-B1] Abas L, Benjamins R, Malenica N, Paciorek T, Wisniewska J, Moulinier-Anzola JC, Sieberer T, Friml J, Luschnig C. Intracellular trafficking and proteolysis of the Arabidopsis auxin-efflux facilitator PIN2 are involved in root gravitropism. Nat Cell Biol. 2006:8(3):249–256. 10.1038/ncb1369 16489343

[koaf056-B2] Abas L, Kolb M, Stadlmann J, Janacek DP, Lukic K, Schwechheimer C, Sazanov LA, Mach L, Friml J, Hammes UZ. Naphthylphthalamic acid associates with and inhibits PIN auxin transporters. Proc Natl Acad Sci U S A. 2021:118(1):e2020857118. 10.1073/pnas.2020857118 33443187 PMC7817115

[koaf056-B3] Bailey MJ, Prehoda KE. Establishment of par-polarized cortical domains via phosphoregulated membrane motifs. Dev Cell. 2015:35(2):199–210. 10.1016/j.devcel.2015.09.016 26481050 PMC4624610

[koaf056-B4] Barbosa IC, Shikata H, Zourelidou M, Heilmann M, Heilmann I, Schwechheimer C. Phospholipid composition and a polybasic motif determine D6 PROTEIN KINASE polar association with the plasma membrane and tropic responses. Development. 2016:143(24):4687–4700. 10.1242/dev.137117 27836964

[koaf056-B5] Barbosa IC, Zourelidou M, Willige BC, Weller B, Schwechheimer C. D6 PROTEIN KINASE activates auxin transport-dependent growth and PIN-FORMED phosphorylation at the plasma membrane. Dev Cell. 2014:29(6):674–685. 10.1016/j.devcel.2014.05.006 24930721

[koaf056-B6] Bassukas AEL, Xiao Y, Schwechheimer C. Phosphorylation control of PIN auxin transporters. Curr Opin Plant Biol. 2022:65:102146. 10.1016/j.pbi.2021.102146 34974229

[koaf056-B7] Blakeslee JJ, Bandyopadhyay A, Lee OR, Mravec J, Titapiwatanakun B, Sauer M, Makam SN, Cheng Y, Bouchard R, Adamec J, et al Interactions among PIN-FORMED and P-glycoprotein auxin transporters in Arabidopsis. Plant Cell. 2007:19(1):131–147. 10.1105/tpc.106.040782 17237354 PMC1820964

[koaf056-B8] Borassi C, Sede AR, Mecchia MA, Salgado Salter JD, Marzol E, Muschietti JP, Estevez JM. An update on cell surface proteins containing extensin-motifs. J Exp Bot. 2016:67(2):477–487. 10.1093/jxb/erv455 26475923

[koaf056-B9] Briggs WR, Christie JM. Phototropins 1 and 2: versatile plant blue-light receptors. Trends Plant Sci. 2002:7(5):204–210. 10.1016/S1360-1385(02)02245-8 11992825

[koaf056-B10] Britz SJ, Galston AW. Physiology of movements in stems of seedling Pisum sativum L. cv. alaska: I. Experimental separation of nutation from Gravitropism. Plant Physiol. 1982:70:264–271. 10.1104/pp.70.5.1401 16662458 PMC1067123

[koaf056-B11] Broer S . Xenopus laevis oocytes. Methods Mol Biol. 2010:637:295–310. 10.1007/978-1-60761-700-6_16 20419442

[koaf056-B12] Day IS, Miller C, Golovkin M, Reddy AS. Interaction of a kinesin-like calmodulin-binding protein with a protein kinase. J Biol Chem. 2000:275(18):13737–13745. 10.1074/jbc.275.18.13737 10788494

[koaf056-B13] Decaestecker W, Buono RA, Pfeiffer ML, Vangheluwe N, Jourquin J, Karimi M, Van Isterdael G, Beeckman T, Nowack MK, Jacobs TB. CRISPR-TSKO: a technique for efficient mutagenesis in specific cell types, tissues, or organs in Arabidopsis. Plant Cell. 2019:31(12):2868–2887. 10.1105/tpc.19.00454 31562216 PMC6925012

[koaf056-B14] Ding Z, Galvan-Ampudia CS, Demarsy E, Langowski L, Kleine-Vehn J, Fan Y, Morita MT, Tasaka M, Fankhauser C, Offringa R, et al Light-mediated polarization of the PIN3 auxin transporter for the phototropic response in Arabidopsis. Nat Cell Biol. 2011:13(4):447–452. 10.1038/ncb2208 21394084

[koaf056-B15] Fastner A, Absmanner B, Hammes UZ. Use of *Xenopus laevis* oocytes to study auxin transport. Methods Mol Biol. 2017:1497:259–270. 10.1007/978-1-4939-6469-7_21 27864772

[koaf056-B16] Fisher CL, Pei GK. Modification of a PCR-based site-directed mutagenesis method. Biotechniques. 1997:23(4):570–574. 10.2144/97234bm01 9343663

[koaf056-B17] Friml J, Wisniewska J, Benkova E, Mendgen K, Palme K. Lateral relocation of auxin efflux regulator PIN3 mediates tropism in Arabidopsis. Nature. 2002:415(6873):806–809. 10.1038/415806a 11845211

[koaf056-B18] Galvan-Ampudia CS, Offringa R. Plant evolution: AGC kinases tell the auxin tale. Trends Plant Sci. 2007:12(12):541–547. 10.1016/j.tplants.2007.10.004 18024140

[koaf056-B19] Gao HB, Chu YJ, Xue HW. Phosphatidic acid (PA) binds PP2AA1 to regulate PP2A activity and PIN1 polar localization. Mol Plant. 2013:6(5):1692–1702. 10.1093/mp/sst076 23686948

[koaf056-B20] Geisler M, Aryal B, di Donato M, Hao P. A critical view on ABC transporters and their interacting partners in auxin transport. Plant Cell Physiol. 2017:58(10):1601–1614. 10.1093/pcp/pcx104 29016918

[koaf056-B21] Geisler M, Blakeslee JJ, Bouchard R, Lee OR, Vincenzetti V, Bandyopadhyay A, Titapiwatanakun B, Peer WA, Bailly A, Richards EL, et al Cellular efflux of auxin catalyzed by the Arabidopsis MDR/PGP transporter AtPGP1. Plant J. 2005:44(2):179–194. 10.1111/j.1365-313X.2005.02519.x 16212599

[koaf056-B22] Graf A, Bassukas AEL, Xiao Y, Barbosa ICR, Mergner J, Grill P, Michalke B, Kuster B, Schwechheimer C. D6PK plasma membrane polarity requires a repeated CXX(X)P motif and PDK1-dependent phosphorylation. Nat Plants. 2024:10(2):300–314. 10.1038/s41477-023-01615-6 38278951 PMC10881395

[koaf056-B23] Grefen C, Donald N, Hashimoto K, Kudla J, Schumacher K, Blatt MR. A ubiquitin-10 promoter-based vector set for fluorescent protein tagging facilitates temporal stability and native protein distribution in transient and stable expression studies. Plant J. 2010:64(2):355–365. 10.1111/j.1365-313X.2010.04322.x 20735773

[koaf056-B24] Grones P, Abas M, Hajny J, Jones A, Waidmann S, Kleine-Vehn J, Friml J. PID/WAG-mediated phosphorylation of the Arabidopsis PIN3 auxin transporter mediates polarity switches during gravitropism. Sci Rep. 2018:8(1):10279. 10.1038/s41598-018-28188-1 29980705 PMC6035267

[koaf056-B25] Guan KL, Dixon JE. Eukaryotic proteins expressed in *Escherichia coli*: an improved thrombin cleavage and purification procedure of fusion proteins with glutathione S-transferase. Anal Biochem. 1991:192(2):262–267. 10.1016/0003-2697(91)90534-Z 1852137

[koaf056-B26] Gupta A, Singh M, Jones AM, Laxmi A. Hypocotyl directional growth in Arabidopsis: a complex trait. Plant Physiol. 2012:159(4):1463–1476. 10.1104/pp.112.195776 22689891 PMC3425191

[koaf056-B27] Haga K, Hayashi K, Sakai T. PINOID AGC kinases are necessary for phytochrome-mediated enhancement of hypocotyl phototropism in Arabidopsis. Plant Physiol. 2014:166(3):1535–1545. 10.1104/pp.114.244434 25281709 PMC4226372

[koaf056-B28] Hajny J, Tan S, Friml J. Auxin canalization: from speculative models toward molecular players. Curr Opin Plant Biol. 2022:65:102174. 10.1016/j.pbi.2022.102174 35123880

[koaf056-B29] Hammes UZ, Murphy AS, Schwechheimer C. Auxin transporters—a biochemical view. Cold Spring Harb Perspect Biol. 2022:14(2):a039875. 10.1101/cshperspect.a039875 34127449 PMC8805647

[koaf056-B30] Han H, Adamowski M, Qi L, Alotaibi SS, Friml J. PIN-mediated polar auxin transport regulations in plant tropic responses. New Phytol. 2021:232(2):510–522. 10.1111/nph.17617 34254313

[koaf056-B31] Hayes S, Pantazopoulou CK, van Gelderen K, Reinen E, Tween AL, Sharma A, de Vries M, Prat S, Schuurink RC, Testerink C, et al Soil salinity limits plant shade avoidance. Curr Biol. 2019:29(10):1669–1676.e4. 10.1016/j.cub.2019.03.042 31056387 PMC6538826

[koaf056-B32] Hilley JL, Weers BD, Truong SK, McCormick RF, Mattison AJ, McKinley BA, Morishige DT, Mullet JE. Sorghum Dw2 encodes a protein kinase regulator of stem internode length. Sci Rep. 2017:7(1):4616. 10.1038/s41598-017-04609-5 28676627 PMC5496852

[koaf056-B33] Humphrey TV, Haasen KE, Aldea-Brydges MG, Sun H, Zayed Y, Indriolo E, Goring DR. PERK-KIPK-KCBP signalling negatively regulates root growth in *Arabidopsis thaliana*. J Exp Bot. 2015:66(1):71–83. 10.1093/jxb/eru390 25262228 PMC4265151

[koaf056-B34] Jensen PJ, Hangarter RP, Estelle M. Auxin transport is required for hypocotyl elongation in light-grown but not dark-grown Arabidopsis. Plant Physiol. 1998:116(2):455–462. 10.1104/pp.116.2.455 9489005 PMC35101

[koaf056-B35] Jonsson K, Ma Y, Routier-Kierzkowska AL, Bhalerao RP. Multiple mechanisms behind plant bending. Nat Plants. 2023:9(1):13–21. 10.1038/s41477-022-01310-y 36581759

[koaf056-B36] Khurana JP, Best TR, Poff KL. Influence of hook position on phototropic and gravitropic curvature by etiolated hypocotyls of *Arabidopsis thaliana*. Plant Physiol. 1989:90(2):376–379. 10.1104/pp.90.2.376 11537453 PMC1061730

[koaf056-B37] Kurihara D, Mizuta Y, Nagahara S, Higashiyama T. ClearSeeAlpha: advanced optical clearing for whole-plant imaging. Plant Cell Physiol. 2021:62(8):1302–1310. 10.1093/pcp/pcab033 33638989 PMC8579160

[koaf056-B38] Kurihara D, Mizuta Y, Sato Y, Higashiyama T. ClearSee: a rapid optical clearing reagent for whole-plant fluorescence imaging. Development. 2015:142(23):4168–4179. 10.1242/dev.127613 26493404 PMC4712841

[koaf056-B39] Lampropoulos A, Sutikovic Z, Wenzl C, Maegele I, Lohmann JU, Forner J. GreenGate—a novel, versatile, and efficient cloning system for plant transgenesis. PLoS One. 2013:8(12):e83043. 10.1371/journal.pone.0083043 24376629 PMC3869738

[koaf056-B40] Lee HJ, Kim HS, Park JM, Cho HS, Jeon JH. PIN-mediated polar auxin transport facilitates root-obstacle avoidance. New Phytol. 2020:225(3):1285–1296. 10.1111/nph.16076 31336402

[koaf056-B41] Lewis DR, Miller ND, Splitt BL, Wu G, Spalding EP. Separating the roles of acropetal and basipetal auxin transport on gravitropism with mutations in two Arabidopsis multidrug resistance-like ABC transporter genes. Plant Cell. 2007:19(6):1838–1850. 10.1105/tpc.107.051599 17557805 PMC1955737

[koaf056-B42] Luschnig C, Gaxiola RA, Grisafi P, Fink GR. EIR1, a root-specific protein involved in auxin transport, is required for gravitropism in *Arabidopsis thaliana*. Genes Dev. 1998:12(14):2175–2187. 10.1101/gad.12.14.2175 9679062 PMC317016

[koaf056-B43] Marhava P, Bassukas AEL, Zourelidou M, Kolb M, Moret B, Fastner A, Schulze WX, Cattaneo P, Hammes UZ, Schwechheimer C, et al A molecular rheostat adjusts auxin flux to promote root protophloem differentiation. Nature. 2018:558(7709):297–300. 10.1038/s41586-018-0186-z 29875411

[koaf056-B44] Noh B, Bandyopadhyay A, Peer WA, Spalding EP, Murphy AS. Enhanced gravi- and phototropism in plant mdr mutants mislocalizing the auxin efflux protein PIN1. Nature. 2003:423(6943):999–1002. 10.1038/nature01716 12827205

[koaf056-B45] Oppenheimer DG, Pollock MA, Vacik J, Szymanski DB, Ericson B, Feldmann K, Marks MD. Essential role of a kinesin-like protein in Arabidopsis trichome morphogenesis. Proc Natl Acad Sci U S A. 1997:94(12):6261–6266. 10.1073/pnas.94.12.6261 9177205 PMC21037

[koaf056-B46] Rahman A, Takahashi M, Shibasaki K, Wu S, Inaba T, Tsurumi S, Baskin TI. Gravitropism of *Arabidopsis thaliana* roots requires the polarization of PIN2 toward the root tip in meristematic cortical cells. Plant Cell. 2010:22(6):1762–1776. 10.1105/tpc.110.075317 20562236 PMC2910985

[koaf056-B47] Rakusova H, Abbas M, Han H, Song S, Robert HS, Friml J. Termination of shoot gravitropic responses by auxin feedback on PIN3 polarity. Curr Biol. 2016:26(22):3026–3032. 10.1016/j.cub.2016.08.067 27773568

[koaf056-B48] Rakusova H, Gallego-Bartolome J, Vanstraelen M, Robert HS, Alabadi D, Blazquez MA, Benkova E, Friml J. Polarization of PIN3-dependent auxin transport for hypocotyl gravitropic response in *Arabidopsis thaliana*. Plant J. 2011:67(5):817–826. 10.1111/j.1365-313X.2011.04636.x 21569134

[koaf056-B49] Saitou N, Nei M. The neighbor-joining method: a new method for reconstructing phylogenetic trees. Mol Biol Evol. 1987:4(4):406–425. 10.1093/oxfordjournals.molbev.a040454 3447015

[koaf056-B50] Sauer M, Kleine-Vehn J. PIN-FORMED and PIN-LIKES auxin transport facilitators. Development. 2019:146(15):dev168088. 10.1242/dev.168088 31371525

[koaf056-B51] Shimada TL, Shimada T, Hara-Nishimura I. A rapid and non-destructive screenable marker, FAST, for identifying transformed seeds of *Arabidopsis thaliana*. Plant J. 2010:61(3):519–528. 10.1111/j.1365-313X.2009.04060.x 19891705

[koaf056-B52] Simon ML, Platre MP, Marques-Bueno MM, Armengot L, Stanislas T, Bayle V, Caillaud MC, Jaillais Y. A PtdIns(4)P-driven electrostatic field controls cell membrane identity and signalling in plants. Nat Plants. 2016:2(7):16089. 10.1038/nplants.2016.89 27322096 PMC4918763

[koaf056-B53] Simura J, Antoniadi I, Siroka J, Tarkowska D, Strnad M, Ljung K, Novak O. Plant hormonomics: multiple phytohormone profiling by targeted metabolomics. Plant Physiol. 2018:177(2):476–489. 10.1104/pp.18.00293 29703867 PMC6001343

[koaf056-B54] Sukumar P, Edwards KS, Rahman A, Delong A, Muday GK. PINOID kinase regulates root gravitropism through modulation of PIN2-dependent basipetal auxin transport in Arabidopsis. Plant Physiol. 2009:150(2):722–735. 10.1104/pp.108.131607 19363095 PMC2689958

[koaf056-B55] Takahashi H, Fujii N, Kamada M, Higashitani A, Yamazaki Y, Kobayashi A, Takano M, Yamasaki S, Sakata T, Mizuno H, et al Gravimorphogenesis of Cucurbitaceae plants: development of peg cells and graviperception mechanism in cucumber seedlings. Biol Sci Space. 2000:14(2):64–74. 10.2187/bss.14.64 11543423

[koaf056-B56] Takahashi H, Jaffe M. Thigmotropism and the modulation of tropistic curvature by mechanical perturbation in cucumber hypocotyls. Physiol Plant. 1990:80(4):561–567. 10.1111/j.1399-3054.1990.tb05679.x

[koaf056-B57] Teale WD, Paponov IA, Palme K. Auxin in action: signalling, transport and the control of plant growth and development. Nat Rev Mol Cell Biol. 2006:7(11):847–859. 10.1038/nrm2020 16990790

[koaf056-B58] Thompson JD, Higgins DG, Gibson TJ. CLUSTAL W: improving the sensitivity of progressive multiple sequence alignment through sequence weighting, position-specific gap penalties and weight matrix choice. Nucleic Acids Res. 1994:22(22):4673–4680. 10.1093/nar/22.22.4673 7984417 PMC308517

[koaf056-B59] Weijers D, Ljung K, Estelle M, Leyser O. Auxin signaling: from synthesis to systems biology. Cold Spring Harbor: Cold Spring Harbor Laboratory Press; 2022.

[koaf056-B60] Weller B, Zourelidou M, Frank L, Barbosa IC, Fastner A, Richter S, Jurgens G, Hammes UZ, Schwechheimer C. Dynamic PIN-FORMED auxin efflux carrier phosphorylation at the plasma membrane controls auxin efflux-dependent growth. Proc Natl Acad Sci U S A. 2017:114(5):E887–E896. 10.1073/pnas.1614380114 28096328 PMC5293077

[koaf056-B61] Willige BC, Ahlers S, Zourelidou M, Barbosa IC, Demarsy E, Trevisan M, Davis PA, Roelfsema MR, Hangarter R, Fankhauser C, et al D6PK AGCVIII kinases are required for auxin transport and phototropic hypocotyl bending in Arabidopsis. Plant Cell. 2013:25(5):1674–1688. 10.1105/tpc.113.111484 23709629 PMC3694699

[koaf056-B62] Yang H, Murphy AS. Functional expression and characterization of Arabidopsis ABCB, AUX 1 and PIN auxin transporters in Schizosaccharomyces pombe. Plant J. 2009:59(1):179–191. 10.1111/j.1365-313X.2009.03856.x 19309458

[koaf056-B63] Ying W, Wang Y, Wei H, Luo Y, Ma Q, Zhu H, Janssens H, Vukasinovic N, Kvasnica M, Winne JM, et al Structure and function of the Arabidopsis ABC transporter ABCB19 in brassinosteroid export. Science. 2024:383(6689):eadj4591. 10.1126/science.adj4591 38513023

[koaf056-B64] Zadnikova P, Petrasek J, Marhavy P, Raz V, Vandenbussche F, Ding Z, Schwarzerova K, Morita MT, Tasaka M, Hejatko J, et al Role of PIN-mediated auxin efflux in apical hook development of *Arabidopsis thaliana*. Development. 2010:137(4):607–617. 10.1242/dev.041277 20110326

[koaf056-B65] Zourelidou M, Absmanner B, Weller B, Barbosa IC, Willige BC, Fastner A, Streit V, Port SA, Colcombet J, de la Fuente van Bentem S, et al Auxin efflux by PIN-FORMED proteins is activated by two different protein kinases, D6 PROTEIN KINASE and PINOID. Elife. 2014:3:e02860. 10.7554/eLife.02860 24948515 PMC4091124

[koaf056-B66] Zourelidou M, Muller I, Willige BC, Nill C, Jikumaru Y, Li H, Schwechheimer C. The polarly localized D6 PROTEIN KINASE is required for efficient auxin transport in *Arabidopsis thaliana*. Development. 2009:136(4):627–636. 10.1242/dev.028365 19168677

