# Supplementary material for: The protein kinases KIPK and KIPK-LIKE1 suppress overbending during negative hypocotyl gravitropic growth in Arabidopsis
Source: Plant Cell. 2025 Apr 22;37(4):koaf056. doi: 10.1093/plcell/koaf056 (PMC12013712; doi:10.1093/plcell/koaf056)
Supplement: koaf056_Supplementary_Data [file koaf056_supplementary_data.zip › Supplementary Figures.pdf]

SUPPLEMENTARY FIGURES

Figure S1

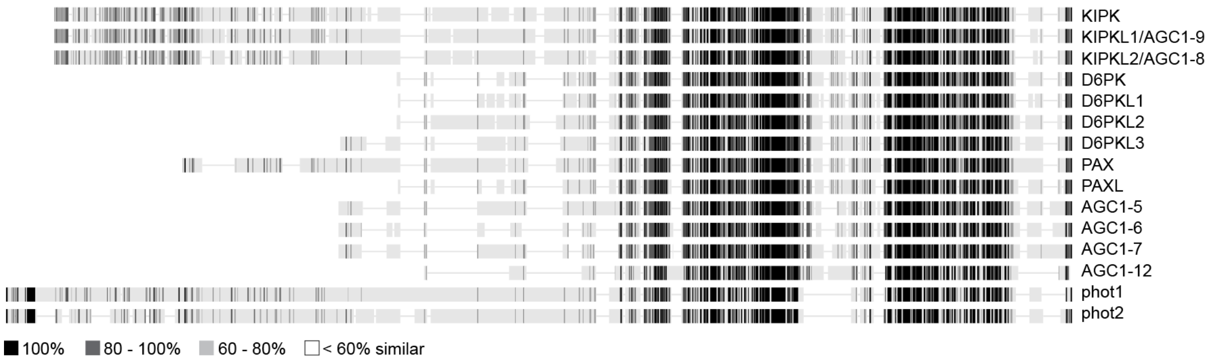

Supplementary Figure S1. Schematic representation of KIPK and KIPKL proteins in the context of *Arabidopsis thaliana* AGC1 and phototropin AGC4 kinases. Schematic representations of KIPK, KIPKL1/AGC1-9, and KIPKL2/AGC1-8, as well as the remaining 10 AGC1 and the two AGC4 blue light receptor serine/threonine kinases phot1 and phot2 from *Arabidopsis thaliana*. AGC1 and AGC4 kinases are two of four subfamilies of the AGCVIII kinase family. The legend specifies the relative amino acid similarity.

Figure S2

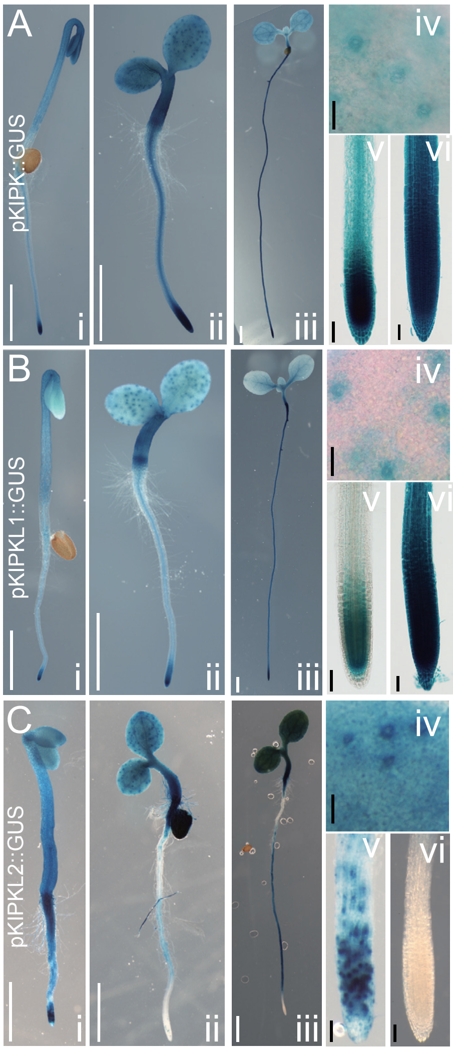

Supplementary Figure S2. Expression analyses using *promoter::GUS* fusions reveal a broad expression pattern of the *KIPK* and *KIPKL* genes. (A) – (C) Photographs of 2.5-day-old dark-grown (i), three- and five-day-old light-grown (ii, iii) seedlings, leaf surfaces with dotted patterns represent stomata staining (iv), as well as root tips of i (v) and iii (vi) from pKIPK::GUS (A), pKIPKL1::GUS (B), and pKIPKL2::GUS (C). Scale bars = 5 mm (i), 3 mm (ii), 3 mm (iii), 40 µm (iv), 50 µm (v), 50 µm (vi).

Figure S3

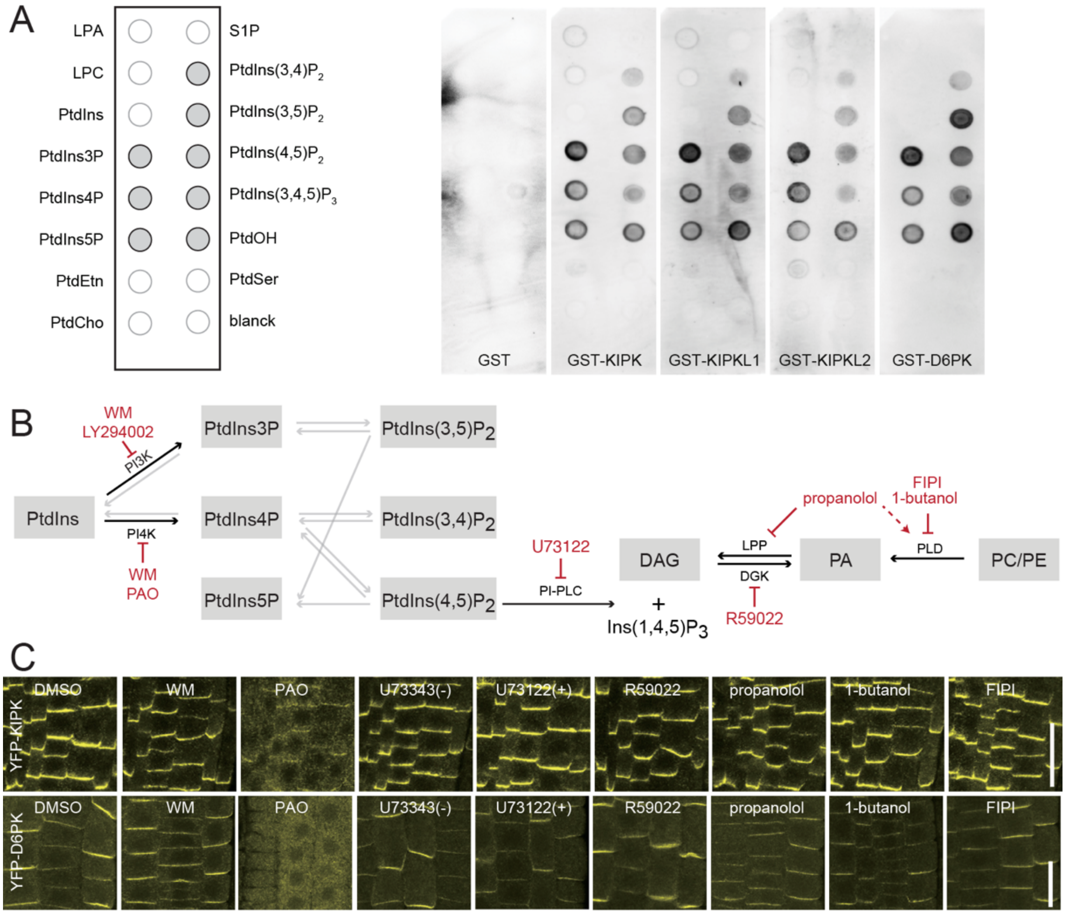

Supplementary Figure S3. KIPK, KIPKL1 and KIPKL2 bind anionic phospholipids. (A) Results of lipid overlay assays with purified GST and GST-tagged kinases as specified. Grey dots in the left panel indicate identities of phospholipids bound by all kinases. **(B)** Schematic overview of the biosynthetic pathways for phosphatidylinositol (PtdIns) and phosphatidic acid (PtdOH) biosynthesis and their chemical inhibitors (red). Black arrows, biosynthetic steps analysed in this study; grey arrows, biosynthetic steps not analysed in this study; dashed arrows, presumed indirect effects of the inhibitors (Meijer and Munnik, 2003; Heilmann, 2009; Potocky et al., 2014; Barbosa et al., 2016; Simon et al., 2016). Abbreviations: DAG, diacylglycerol; DGK, DAG KINASE; LPA, lysophosphatidic acid; LPC, lysophosphatidylcholine; LPP, LIPID PHOSPHATE PHOSPHATASE; PAO, phenylarsine oxide; PI-PLC, PI-SPECIFIC PHOSPHOLIPASE C; PLD, PHOSPHO-LIPASE D; WM, Wortmannin; PtdCho, phosphatidylcholine; PtdEtn, phosphatidylethanolamine; PtdIns, phosphatidylinositol and its mono-/bis-/tris-phosphates; PtdSer, phosphatidylserine; S1P, sphingosine-1-phosphate. **(C)** Representative confocal images of epidermal cells expressing YFP-KIPK or YFP-D6PK after mock treatment (30 min) and treatments (30 min) with the specified inhibitors: 0.1% DMSO, 33 µM WM (Wortmannin), 30 µM PAO (phenylarsenic oxide), 5 µM U73343 (−) inactive and U73122 (+) active analogues, 50 µM R59022, 50 µM propanolol, 0.8% 1-butanol, 1 µM FIPI (5-fluoro-2-indolyl des-chlorohalopemide). The images of the DMSO control and the PAO treatment are identical to the ones shown in Figure 1C. Scale bars for all images = 20 µm.

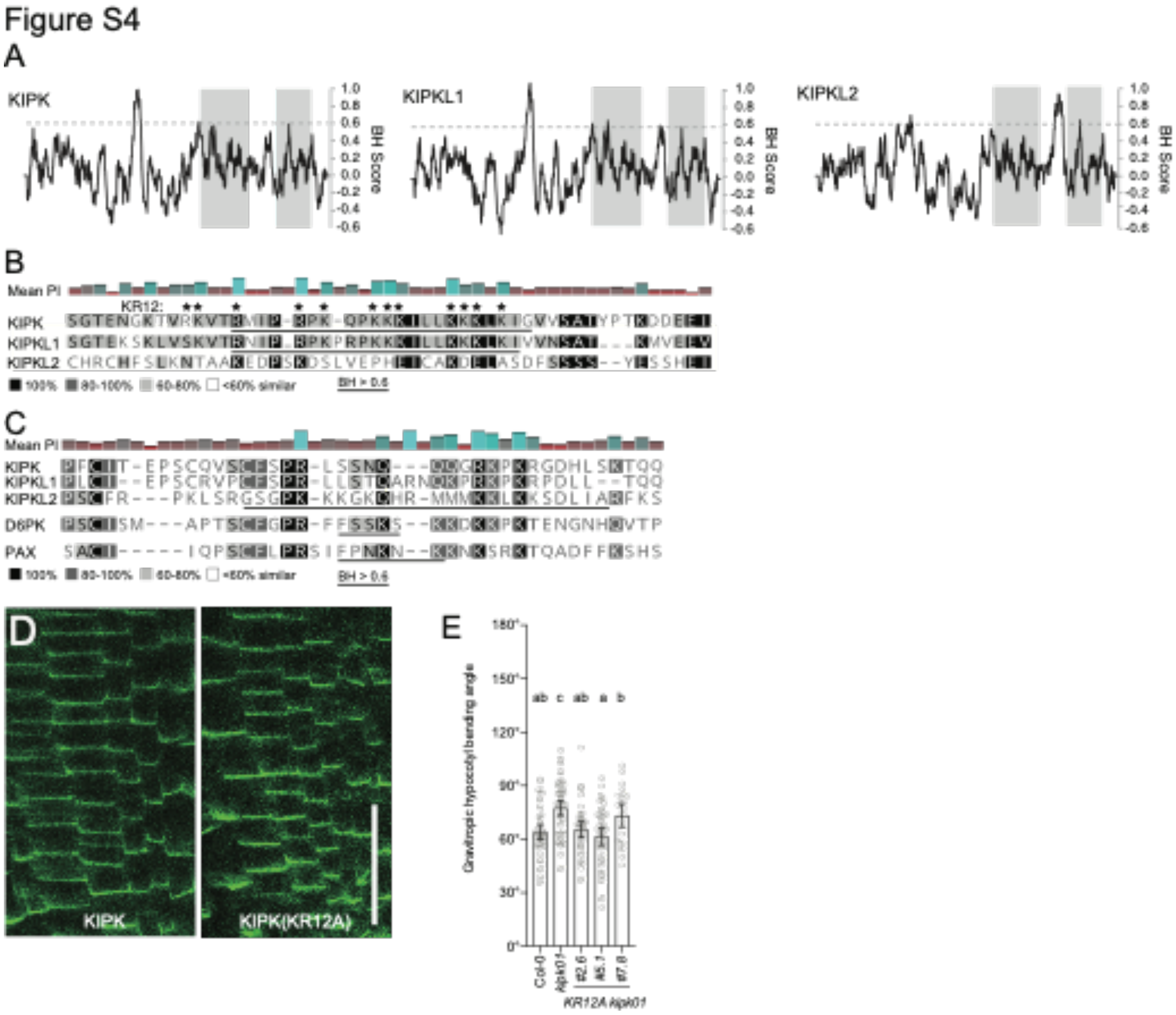

Supplementary Figure S4. A polybasic region in the N-terminus of KIPK is dispensable for plasma membrane interactions. (A) Basic hydrophobicity (BH) profiles of KIPK, KIPKL1, and KIPKL2. A BH score greater than 0.6 had previously been shown to be a good predictor for interactions with phospholipids (Bailey and Prehoda, 2015). **(B)** and **(C)** Protein sequence alignments of the basic hydrophobic regions (BH > 0.6) and a display of the mean isoelectric points (mean pI) of the N-terminal regions of the specified proteins (B) or from their middle domains (C). Asterisks mark the 12 K and R residues that were mutagenized to A to obtain KIPK(KR12A). The legends specify the relative amino acid similarity. **(D)** Representative confocal images of root epidermis cells expressing eGFP-KIPK or eGFP-KIPK(KR12A) in the *kipk01* mutant. Scale bar for both images = 25 µm. **(E)** Graph displaying the average and 95% confidence interval, as well as the individual data points from a negative hypocotyl gravitropism experiment. n > 23 seedlings. Statistical significance was assessed using one-way ANOVA, followed by Dunnett's T3 post hoc test for multiple comparisons. Different letters indicate significant differences between groups at $p < 0.05$.

Figure S5

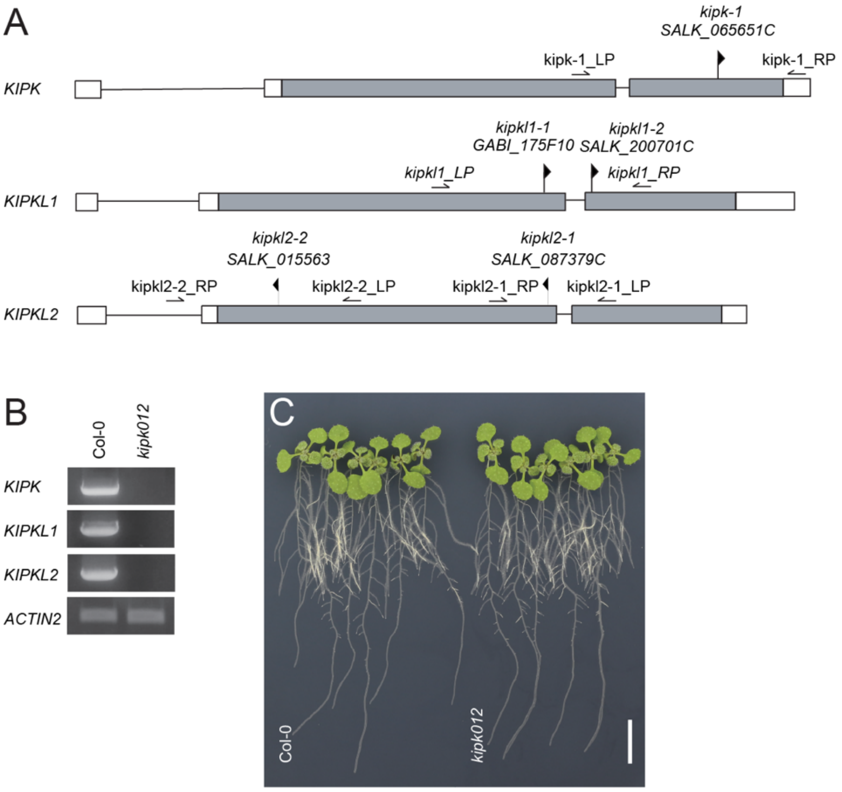

Supplementary Figure S5. Mutants of *KIPK* and *KIPKLs* do not display apparent growth defects. (A) Schematic representation of *Arabidopsis thaliana KIPK*, KIPKL1, and *KIPKL2* genes with positions of T-DNA insertions in their mutant alleles. The arrowheads indicate the position and the direction of the T-DNA insertion as deposited at the SIGNAL web resource (signal.salk.edu). Left (LP) and right (RP) border primers for genotyping and RT-PCR are shown by arrows. **(B)** Results from reverse transcription RT-PCR analyses using the primers specified in (A) of the *kipk012* mutant with the alleles *kipk-1, kipkl1-1*, and *kipkl2-1*. *ACTIN2* serves as a control gene transcript. **(C)** Representative photograph of 14-day-old light-grown wild-type and *kipk012* plants. Scale bar = 1 cm.

Figure S6

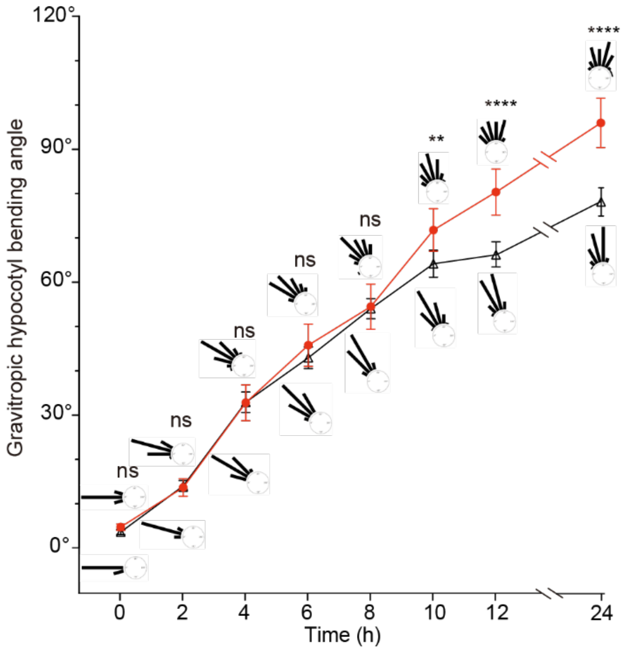

Supplementary Figure S6. *kipk01* mutants are defective in bending during the later stages of the bending response. Graph displaying the average and 95% confidence interval from a negative hypocotyl gravitropism experiment with a time resolution of wild type (black) and *kipk01* mutants (red). $n \geq 60$ seedlings. Rose diagrams with angle distributions, as well as results from a Welch's t-test from a comparison between wild-type and mutant samples of a given time point, are displayed for each time point: ** $p < 0.01$; **** $p < 0.0001$; ns, not significant.

Figure S7

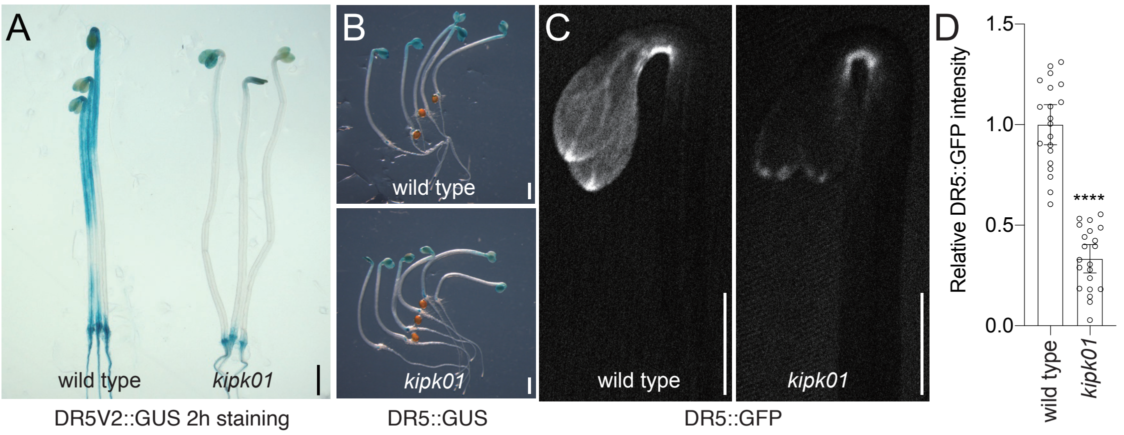

Supplementary Figure S7. *kipk01* mutants display reduced auxin responses. (A) – (C) Representative photographs (A, B) and confocal microscopy images of 3.5-day-old (A) or 3-day-old (B, C) dark-grown seedlings expressing the GUS (A, B) or GFP (C) reporters, as specified, after 2 h and 4 h GUS staining (A, B) or confocal imaging (C). Scale bars = 1 mm (A, B) and 500 µm (C). **(D)** Graph displaying the average and 95% confidence interval, as well as the individual data points of the relative DR5::GFP intensity measured in cotyledon together with the apical hook region of *kipk01* and wild-type seedlings. n = 20; Welch's t-test, ****, $p < 0.0001$.

Figure S8

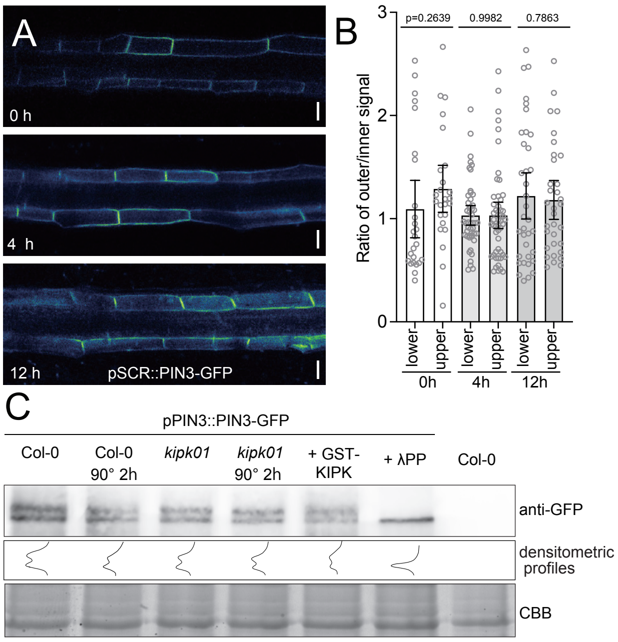

Supplementary Figure S8. PIN3-GFP lateral distribution is stable during the gravitropism response. (A) Representative confocal microscopy images of hypocotyl sections of three-day-old dark-grown seedlings expressing pSCR::PIN3-GFP at time points 0, 4, and 12 hrs after gravistimulation. Scale bars = 20 µm. n > 10 seedlings. **(B)** Graph displaying the average and 95% confidence interval, as well as the individual data points of ratios between the outer and inner PIN3-GFP signal during gravitropic hypocotyl bending at time points 0, 4, and 12 hrs after gravistimulation. n > 24 cells. A Welch's t-test result is displayed on top of each bar. **(C)** Representative western blot with anti-GFP antibody for the detection of PIN3-GFP before and 2 hrs after gravistimulation in the wild type (Col-0), kipk01, and after the addition of purified recombinant GST-KIPK or λ phosphatase (λPP) from four-day-old dark-grown seedlings expressing pPIN3::PIN3-GFP or the non-transgenic wild type. The upper band corresponds to a phosphorylated form of PIN3-GFP, as revealed by the absence of this band after phosphatase treatment. Densitometric profiles (middle panel) do not suggest major changes in the abundance of PIN3-GFP between the wild type (Col-0) and *kipk01*, or in the phosphorylated PIN3-GFP form between the genotypes and treatments or following gravistimulation. CBB, Coomassie Brilliant Blue-stained gel section, loading control.

Figure S9

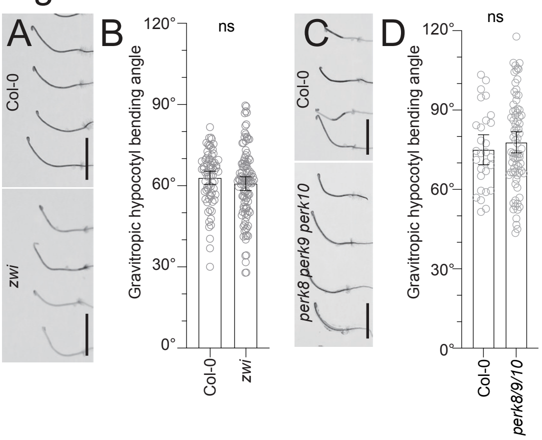

Supplementary Figure S9. *ZWI*, *PERK8*, *PERK9*, and *PERK10* are not required for gravitropic hypocotyl bending. (A) and **(C)** Representative photographs of three-day-old dark-grown seedlings of the specified genotypes 24 hours after reorientation by 90°. Scale bars = 1 cm. **(B)** and **(D)** Graphs displaying the average and 95% confidence interval, as well as the individual data points ($n \geq 29$) from a negative hypocotyl gravitropism experiment as shown in (A) and (B). Welch's t-test: $p > 0.05$, ns, not significant.
